# Effectiveness of the Self-Directed mHealth Exercise Intervention re.flex in Patients With Knee Osteoarthritis: Randomized Controlled Trial

**DOI:** 10.2196/71558

**Published:** 2025-10-09

**Authors:** Valerie Dieter, Peter Martus, David Seißler, Lina Maria Serna-Higuita, Pia Janssen, Inga Krauss

**Affiliations:** 1 Department of Sports Medicine, Medical Clinic, University Hospital Tübingen, Hoppe-Seyler-Str. 6, Tübingen, 72076, Germany, 49 70712986477; 2 Interfaculty Research Institute for Sports and Physical Activity, Tübingen, Germany; 3 Department for Clinical Epidemiology and Applied Biostatistics, Medical Clinic, University Hospital Tübingen, Tübingen, Germany; 4 fbeta GmbH, Berlin, Germany

**Keywords:** digital health, mHealth, exercise intervention, knee osteoarthritis, knee pain, physical function

## Abstract

**Background:**

About 1 in 2 patients with knee osteoarthritis (OA) receives a referral or recommendation for exercise. Digital health applications could counteract this undersupply.

**Objective:**

We aimed to investigate the effectiveness of a 12-week self-directed mobile health exercise intervention (re.flex) when used in addition to usual care compared to a control group receiving usual care only on pain reduction and improvement in physical function in patients with knee OA.

**Methods:**

This monocentric, 2-arm, randomized controlled parallel-group trial included patients from Germany with moderate to severe knee OA. Participants were mainly recruited via newspapers. Randomization was 1:1 into an intervention group (re.flex+usual care) and a control group (usual care) using computer-generated blocks. Participants were unmasked to group assignment. The re.flex group conducted a 12-week self-directed app-based and sensor-assisted exercise program with 3 sessions per week in addition to usual care. Primary outcomes were OA-specific knee pain and physical function (using the subscales pain and activities in daily living of the Knee Osteoarthritis Outcome Score, 0‐100) at 3 months. Secondary outcomes included adherence and safety. Multiple imputation was used to account for missing data. Intervention effects were calculated using a baseline-adjusted analyses of covariance (ANCOVA). Bonferroni correction with an alpha level of .025 was applied.

**Results:**

Between January 25, 2023, and August 11, 2023, a total of 195 participants were enrolled. Of them, 98 participants were allocated to re.flex, and 97 participants to usual care. The primary analysis included 194 participants. The mean age was 61.9 (SD 7.7) years, and the majority were female (132/194, 68%). Pain reduction was significantly larger in re.flex than in usual care, with an adjusted mean difference between study groups of 4.8 (95% CI 0.7-8.9; *P*=.02; Cohen *d*=0.35) points. Improvement in physical function was not statistically significant (beta coefficient [β]=3.9 points, 95% CI 0.0-7.9, *P*=.049). A total of 12 adverse events were linked to re.flex, none of which were serious. Participants adhered to 77% (2705/3528) of all scheduled exercise sessions.

**Conclusions:**

The self-directed sensor-based mobile health exercise intervention re.flex demonstrates superiority over usual care for pain reduction and justifies this kind of intervention as an alternative exercise delivery mode for patients with knee OA.

## Introduction

Osteoarthritis (OA) is a degenerative joint disease and a leading cause of adult chronic pain and long-term disability. It most commonly affects the knee, hip, and hand joints [1,2]. Higher prevalence rates are reported for higher age, female sex, and number of comorbidities, for example, obesity [3]. According to the German Gesundheit in Deutschland aktuell (GEDA) 2014/2015-European Health Interview Survey (EHIS), 17.9% of German adults have been diagnosed with OA. Among the age group of >65 years, almost half of the female and one-third of the male German population are affected [4].

Clinical guidelines worldwide outline exercise as a first-line treatment in patients with knee OA [5]. However, only 50% of patients receive recommendations for physiotherapy and only 36% for therapeutic exercise [6,7]. There is an urgent need to investigate additional ways for guiding exercise in these patients. To this end, digital health technologies seem promising, as they are widely available and allow users to exercise independent of location and time. Digital health is an umbrella term for the field of eHealth, mobile health (mHealth), and telehealth. They enable the delivery of health services by integrating digital devices and tools that improve the accessibility and efficiency of health care [8]. In general, health care interventions delivered by digital health technologies can be classified into three main categories: (1) self-directed eHealth and mHealth interventions (eg, web or mobile apps [9-11]), (2) synchronous [12,13] or asynchronous [14] supervised eHealth and mHealth interventions (eg, videoconferencing, chat messages in combination with mobile apps, and phone calls), and (3) blended care approaches [15,16] (hybrid format combining an in-person supervised intervention guided by a health care professional in individual treatment or group sessions blended with a self-directed or remotely supervised eHealth or mHealth intervention).

The results of a qualitative study indicate that patients with knee OA mostly seem to have positive experiences with and attitudes toward the use of self-directed eHealth exercise interventions [17]. However, people prefer different levels of human support, illustrating that there is no one-size-fits-all solution for implementing eHealth exercise interventions in OA care [17]. Thus, a variety of delivery modes could be offered, depending on the patient’s individual health care needs, preferences, and individual context factors. To better ensure tailoring an appropriate level of input, the implementation of digital interventions could be integrated into a stepped care model [18]. This would entail the initial provision of self-directed offers, with the possibility of progressing to a more supervised option in case no meaningful improvements were achieved in previous steps [17,19]. The results of recent meta-analyses demonstrated that eHealth exercise interventions have a beneficial effect on pain and physical function in patients with knee OA. The effects are comparable to those of nondigital, face-to-face interventions and superior to usual care [20,21].

To our knowledge, no systematic review or meta-analysis has directly compared treatment effects between supervised and self-directed eHealth exercise interventions for knee OA. However, the possibility of human supervision and interaction in digitally delivered exercise interventions is considered to positively influence motivation for and adherence to exercise and may improve clinical outcomes [22,23]. The effectiveness of supervised exercise interventions can be attributed to specific factors, including the high quality with which exercise programs are performed (eg, instructional guidance, technical execution of exercises, higher intensity, more comprehensive introduction of individualization and progression principles, and direct feedback) and giving patients the confidence to perform the exercises correctly [24-26]. Thus, incorporating health care professional input may foster acceptance and engagement [17]. Nevertheless, this does not fully address the issue of limited health care accessibility. In the absence of human interaction, the use of app features, such as biofeedback (eg, movement sensors) or self-monitoring, in-depth guidance on how to exercise correctly (eg, exercise videos, instructions, repetition, and set numbers), and SMS text messages or push notifications to remind users of upcoming exercise sessions may provide a similar level of assistance [17,27,28].

As previously stated, self-directed digital care solutions have the potential to address the insufficient provision of exercise as a first-line treatment in knee OA. However, patient benefit and reimbursement strategies are prerequisites for a successful implementation into clinical routine. In Germany, the Digital-Care-Law (Digitale-Versorgung-Gesetz, DVG) allows physicians to prescribe digital health applications (Digitale Gesundheitsanwendung, DiGA) registered in the “DiGA directory” [29].

This randomized controlled trial aimed to examine the effectiveness of the sensor-assisted mHealth exercise intervention (re.flex). The confirmatory study aims to test the primary hypotheses concerning the superiority of the intervention (re.flex+usual care) over usual care in reducing pain and improving physical function.

## Methods

### Study Design and Participants

The study was conducted as a monocentric randomized controlled superiority trial as outlined in the study protocol [30]. The study report follows the CONSORT-EHEALTH (Consolidated Standards of Reporting Trials of Electronic and Mobile Health Applications and Online Telehealth) checklist [31]. Participants were recruited via advertisements in newspapers, digital media, referrals from physicians, and 2 information events about OA. Individuals aged 18 years or older with (1) a self-reported tibiofemoral knee OA diagnosed by a physician, (2) a self-reported Knee Osteoarthritis Outcome Score (KOOS) of the subscale pain ≤60 points during the screening process, and (3) a mobile electronic device with an iOS or Android operating system were eligible for inclusion. Participants were excluded (1) if they had a history of knee joint replacement or osteotomy on the signal joint, (2) if contraindications were present that precluded the safe execution of exercise without supervision, (3) if they were currently treated by a physician or physiotherapist for any complaints of the lower extremity or lower back other than knee OA, (4) if they had been conducting regular structured strengthening exercises for the lower extremities more than once a week during the past 6 months, or (5) if they had insufficient German language proficiency. Computer or internet literacy was not an eligibility criterion. Interested persons were screened by phone. Final inclusion or exclusion took place at the University Hospital Tübingen in the context of the medical examination at baseline.

### Randomization and Masking

Participants were randomly assigned (1:1) to re.flex (see intervention section) in addition to usual care (intervention group) or usual care (control group). Randomization lists were created for each of the 8 combinations of the stratification factors etiology (primary and secondary OA), medication (regularly, none, or sporadic), and laterality of the disease (one-sided, both-sided) using computer-generated random numbers (0, 1) with varying block lengths. The randomization lists were transferred to the data management system SecuTrial (Interactive Systems). Sequence generation and concealment were done by an employee (RB) who was not involved in the conduction and assessment of the study. By using randomization via the software SecuTrial (Interactive Systems), allocation concealment was ensured. Participants were not randomized in case of exclusion before completion of the examination and assessments at baseline. This trial was not blinded for participants. Baseline assessments took place before randomization, and assessors for the 30-Second Chair Stand Test were blinded toward group allocation for follow-up assessments. However, the assessment of patient-reported outcome measures (PROMs) was at risk for reporting bias. The statistician was blinded toward the primary outcomes (except for the jump to reference imputation, where the blinding was not possible). For further analyses, the statistician was unblinded, as treatment allocation was obvious (eg, usability, logfiles).

### Intervention

**Figure 1.**
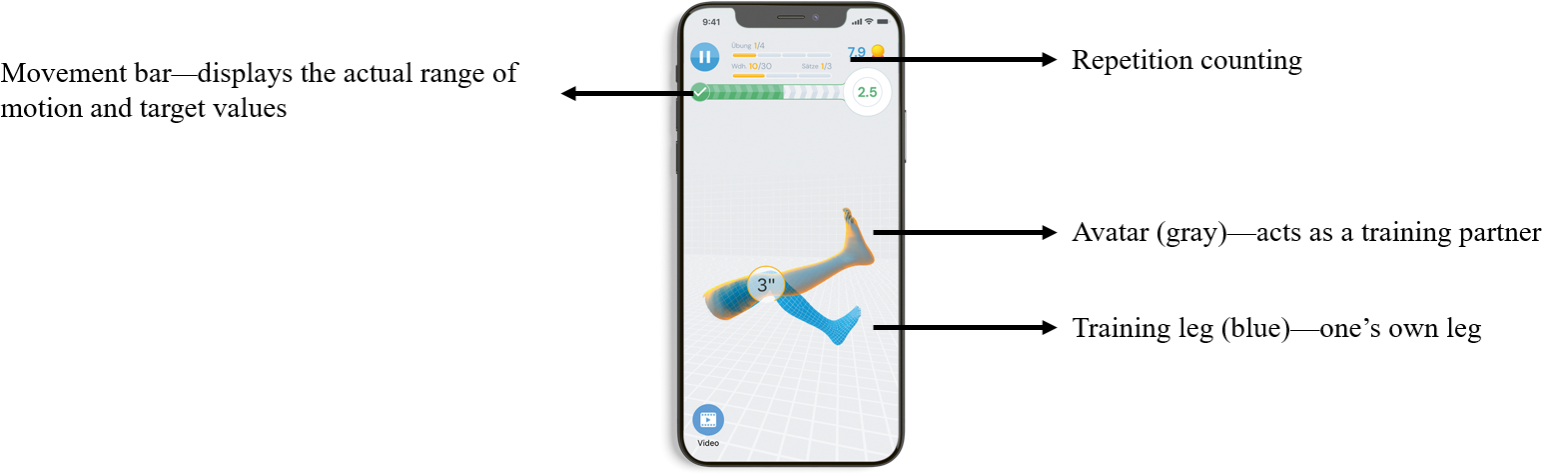
Screenshot with features of the re.flex app.

The intervention group received re.flex (2019, Kineto Tech Rehab SRL, Romania), a 3-month training program with exercises guided by use of an app and 2 wearable motion sensors (3-axis gyroscope, L 43 mm x W 24 mm x H 12 mm, weight 16 grams, Bluetooth Low Energy) attached proximally and distally to the OA-affected knee joint (refer to Multimedia Appendix 1). The primary focus of the intervention was to strengthen knee extensors, knee flexors, and hip abductors. Further exercises were aimed at joint mobilization, muscle stretching, and balance training. Different types (eg, open and closed kinetic chain), exercise variations (eg, short or long lever arms, elastic resistance bands), and poses (supine, sitting, and standing) were used to allow progression of training loads. In addition, the user could choose one of two difficulty levels for strengthening exercises, with the lower one set as default. Details on the progressively designed program with dosage principles and objectives are described in the Multimedia Appendix 1. All training sessions were self-directed and conducted at home. At baseline, the patient was introduced into downloading, logging into the free user account, and usage of the app, sensors, and dosage principles by the study staff. The app provided the exercises with text descriptions and videos. Using biofeedback provided by the motion sensors, the patient was asked to align his virtual limb to the target condition displayed with another avatar in order to control movement execution, predefined range of motion, movement velocity, and number of repetitions. Visual and auditory feedback was further provided by a movement bar, a real-time rating of movement quality, and an auditory signal whenever reaching the end position of a movement. If an exercise was not performed correctly, verbal instructions were given. Further app features allowed users to pause or skip exercises, rate pain and perceived exertion during exercise sessions, remind users of upcoming training sessions via push notification, monitor training progress with statistics, and allow users to contact the app provider in case of technical issues via an app messenger service. A recommendation to pause training and to contact the physician in charge was provided if the maximum pain level was entered. In the context of the study, medical issues (adverse events [AEs]) were reported to the study personnel via email or phone call. In addition, patients did receive information on how to deal with increasing pain during or after exercising, if applicable. All information given at baseline was provided orally and written on a fact sheet and an instruction manual. Details and screenshots of the app features are displayed in Figure 1 and the Multimedia Appendix 1. Participants in the control group did not receive any study intervention but were allowed to use usual care (for details of how usual care was defined, refer to the study protocol [30]), in correspondence to the re.flex group. Furthermore, participants were given the opportunity to use re.flex after study completion.

### Outcomes

Data were collected at baseline (t_0_) and 3 months (t_1_). PROMs were assessed web-based (Questback GmbH, Germany), and the 30-Second Chair Stand Test was conducted on-site. Concomitant care was additionally assessed web-based 4 and 8 weeks after baseline. App log files were read out after 3 months. Primary outcomes were baseline-adjusted follow-up scores (t_1_) of pain and physical function, measured with the KOOS subscales pain and activities in daily living, each on a scale of 0‐100, worst to best.

Secondary outcomes included: (1) KOOS subscales, such as symptoms, function in Sport and Recreation, and knee-related quality of life (QoL); (2) patient global assessment (PGA) of knee OA; (3) Health-related quality of life (HrQoL; Veterans RAND 12-Item Health Survey [VR-12]) with the mental component scores (MCS) and physical component scores (PCSs); (4) subjective change in health status along the study period in general, for pain, and walking; (5) the mHealth App Usability Questionnaire (MAUQ, self-translated into German language); (6) patient satisfaction with the app and results of the treatment; and (7) the 30-Second Chair Stand Test. Additional outcomes included the evaluation of log files (adherence, active time, rating of perceived exertion, and pain), AEs occurring during the study period, and any concomitant care as a potential confounder. Full details on descriptive and outcome measures are in the study protocol [30] and the Multimedia Appendix 1.

### Statistical Analysis

The extant literature has reported minimal clinically important differences (MCIDs) between 5.5 and 8.7 points for nonsurgical treatment strategies in patients with knee OA [32,33]. Accordingly, this study was powered on an MCID of 5 points (0‐100) between the intervention and control group for the first primary outcome “KOOS subscale pain,” assuming the pooled SD of differences to baseline according to the pilot study [34]. This led to an effect size of 0.5. Therefore, an estimated sample size of 156 participants was required to detect a MCID of 5 points between groups for the primary outcomes, with a type I error of 0.025 (2-sided, Bonferroni correction for 2 confirmatory outcomes and success of the study if at least 1 outcome was significant in favor of the intervention) and a power of 80%. For adjustment of baseline, etiology, medication, and laterality, 4 additional df are spent, and the sample size was increased to 160. Considering a dropout rate of ~20%, we aimed to recruit 200 participants (100 per group) into the study. The sample size was determined using the software nQuery+ nTerim (Statistical Solutions Ltd) release 4.0. For additional information regarding the sample size, refer to the published study protocol [30] or Multimedia Appendix 1.

All statistical analyses have been done using the software SPSS (version 27.0; IBM Corp) and R (version 4.1.2; R Foundation for Statistical Computing). No interim analysis was performed. There was no external data monitoring committee for this study.

The primary analysis population was the intent-to-treat (ITT) population. This population includes all patients who contributed at least baseline values of the primary outcomes. Multiple imputation (MI) on score level was applied under the assumption of data missing at random to participants who dropped out or did not contribute measurements of the primary outcomes for other reasons. Baseline measurements were included as predictors. The “jump to reference” method was used for the primary analysis, that is, patients who dropped out were assigned to the control arm in the imputation model but not in the analysis model (ITT). This leads to a shrinkage of the intervention effect and is thus a conservative approach. The 2 primary outcomes (KOOS subscale pain and KOOS subscale activities in daily living at 3 months) were evaluated using a baseline-adjusted analyses of covariance (ANCOVA) with the primary factor “intervention” (study arm). The 3 stratification variables (etiology, medication, and laterality) were included as additional fixed factors (binary coding). For the main results, parameter estimates (beta coefficients, unstandardized differences), *P* values, 2-sided 95% CIs, and effect measures (Cohen *d* calculated as mean difference divided by SD of differences) were calculated.

Sensitivity analyses included an analysis with the ITT using MI without jump to reference, a complete case analysis, a per-protocol analysis (all participants using the app until the last 2 weeks of the intervention phase with an overall adherence rate of at least 80% of the scheduled exercise sessions, applicable only in the intervention group), and last observation carried forward (LOCF) in the ITT. Continuous secondary outcomes were analyzed using the same statistical methods as used for the primary outcomes. However, these were secondary analyses, which may not be interpreted as confirmatory.

Exploratory subgroup analyses for the 2 primary outcomes were performed for the stratification factors (etiology, medication, and laterality), patient age (40‐55 years, 55‐64 years, and ≥65 years), and patient sex. Additional subgroup analyses were performed in the case of frequent or differential occurrence of concomitant care between study groups. In this context, nonsteroidal anti-inflammatory drugs, physiotherapy, and insoles qualified for subgroup analysis, with medication only being considered if taken on a regular basis (weekly or daily). Subgroup analyses were considered if interaction terms of the study arm and the respective subgroup variable were statistically significant.

A responder analysis was conducted using transition scales for subjective ratings of overall change, change in pain, and change in function with a 5-point Likert scale (much worse, somewhat worse, unchanged, somewhat better, and much better). In this analysis, the categorical values of response scales were dichotomized into the 2 groups “improved” (somewhat better or much better) and “not improved” (unchanged, somewhat worse, or much worse). Missing values of dichotomized data were imputed with jump to reference and analyzed using logistic regression models with linear predictors identical to the model of the primary analysis. Odds ratio obtained from logistic regression analysis, success ratio (SR) according to the calculation of “risk ratio,” and numbers needed to treat (NNT) obtained from descriptive analyses were used as effect measures for each scale separately.

### Ethical Considerations

All participants provided written informed consent. Ethical approval was obtained from the ethics committee of the University Hospital Tübingen (688/2022BO2). The participants were given a study ID number. Identifiable information was stored on password-protected servers. There was no compensation for participation in the study. Study materials were provided to participants for free. The study was registered in the German clinical trial register (DRKS00030932).

## Results

### Participant Flow and Baseline Data

**Figure 2.**
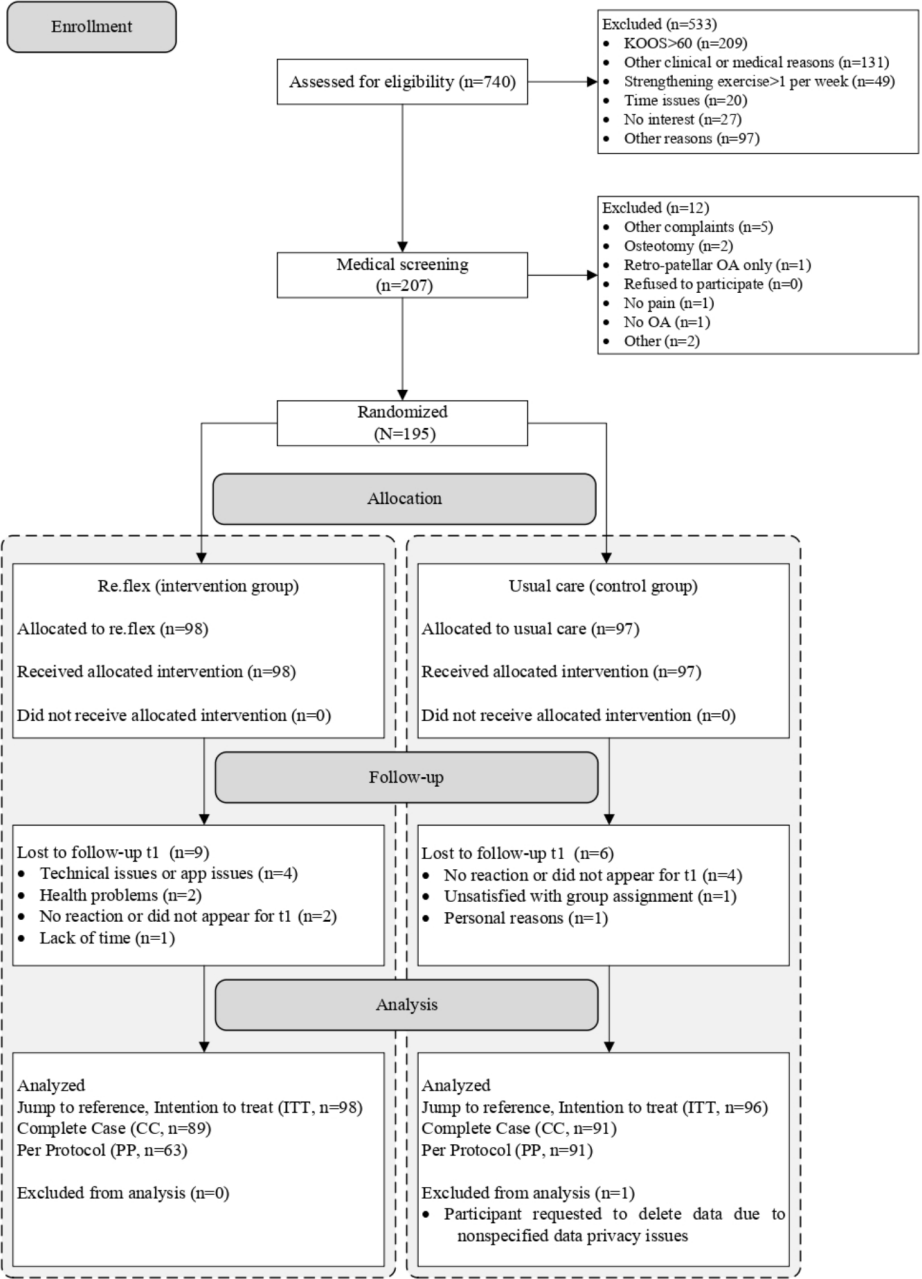
Participant flowchart. KOOS: Knee Osteoarthritis Outcome Score; OA: osteoarthritis.

Between January 25, 2023, and August 11, 2023, 195 participants were enrolled. Thereof, 98 participants were allocated to the re.flex group and 97 participants to usual care. Loss to follow-up was 9.2% (9/98) in the re.flex group and 6.2% (6/97) in usual care. One participant had to be excluded from the analyses due to the participant’s request to delete all his data. Details on participant flow are displayed in Figure 2.

The trial recruitment was stopped once the planned number of evaluable participants was reached, as the dropout rate was lower than assumed (<20%). Baseline characteristics and baseline values of primary and secondary outcomes of participants did show comparable distributions between study groups (Table 1 and Multimedia Appendix 1).

**Table 1.** Baseline characteristics of participants by study group^a^.

Baseline characteristics		re.flex group (n=98)	Usual care group (n=96)
Demographics, anthropometry, and socioeconomics			
Age (years), mean (SD)		61.6 (8.0)	62.1 (7.5)
Sex, n (%)			
Female		70 (71)	62 (65)
Male		28 (29)	34 (35)
BMI (kg/m²), median (IQR)		28.4 (26.8-32.2)	27.8 (25.0-31.4)
German nationality, n (%)		95 (97)	93 (97)
Life situation, n (%)			
Working		61 (62)	48 (50)
Retired		34 (35)	43 (45)
Others		3 (3)	5 (5)
Education, n (%)			
Low (<10 years)		8 (8)	9 (9)
Middle (≥10 years)		36 (37)	34 (35)
High (university entrance qualification)		49 (50)	48 (50)
Others		5 (5)	5 (5)
Housing, n (%)			
Together with partner		83 (85)	82 (85)
Alone		14 (14)	13 (14)
Others		1 (1)	1 (1)
Stratification factors			
Laterality, n (%)			
One-sided knee OA^b^		37 (38)	36 (38)
Both-sided knee OA^b^		61 (62)	60 (62)
Etiology, n (%)			
Primary knee OA^b^		84 (86)	83 (87)
Secondary knee OA^b^		14 (14)	13 (13)
Medication (knee OA–related)^b^, n (%)			
No or sporadic		91 (93)	90 (94)
Regularly		7 (7)	6 (6)
Anamnesis			
Time since diagnosis of knee osteoarthritis (months), median (IQR)		65 (24-120)	61 (24-120)
Signal joint – Knee, n (%)			
Right		52 (53)	57 (59)
Left		46 (47)	39 (41)
History of injury, n (%)			
Ligament injury		22 (22)	21 (22)
Meniscus damage		50 (51)	57 (59)
No previous history		31 (32)	25 (26)
Comorbidities, n (%)			
Heart disease		6 (6)	8 (8)
Hypertension		35 (37)	33 (34)
Stroke		0 (0)	0 (0)
Circulatory disorder		2 (2)	1 (1)
Lung disease		7 (7)	5 (5)
Diabetes		3 (3)	3 (3)
Kidney disease		1 (1)	0 (0)
Neurological disease		0 (0)	1 (1)
Liver disease		1 (1)	2 (2)
Cancer		6 (6)	3 (3)
Depression		4 (4)	4 (4)
Back injury		14 (15)	8 (8)
Activity and training variables			
Physical activity (per week), n (%)			
No		3 (3)	2 (2)
30 minutes		9 (9)	5 (5)
1 hour		20 (20)	20 (21)
2 hours		20 (20)	19 (20)
More than 2 hours		46 (47)	50 (52)
Previous experience with strength training, n (%)			
Very high		2 (2)	11 (11)
High		22 (22)	16 (17)
Medium		49 (50)	43 (45)
Low		22 (22)	17 (18)
Very low		3 (3)	9 (9)
Previous experience with hip or knee group training, n (%)			
Yes		6 (6)	9 (9)
No		92 (94)	87 (91)
Primary outcomes, mean (SD)			
Pain (0-100^c^)		53.9 (14.4)	54.9 (12.8)
Physical function (0-100^d^)		66.7 (15.1)	67.7 (15.2)
Others, mean (SD)			
Technical affinity (–2 to 14^e^)		7.4 (2.1)	7.7 (1.9)
Outcome expectation (5-20^f^)		14.4 (3.3)	14.3 (2.9)
Fear of Movement (6-24)^g^		9.8 (3.3)	9.5 (2.6)

a Data are mean (SD), n (%), or median (IQR). Some percentages might not add to 100% due to rounding.

b OA: osteoarthritis.

c Pain was assessed with the Knee Osteoarthritis Outcome Score (KOOS) subscale pain (0-100, worst to best) using a 5-point Likert scale.

d Physical function was assessed with the Knee Osteoarthritis Outcome Score (KOOS) subscale Activities in Daily Living (ADL) (0-100, worst to best) using a 5-point Likert scale.

e Technical affinity was assessed with the Technical Affinity – Electronic Devices (TA-EG) questionnaire using a 5-point Likert scale with options of “completely applicable,” “rather applicable,” “partly,” “rather not applicable,” and “not applicable at all.” Higher scores reflect a better technical affinity.

f Outcome expectation was assessed with the Expectation for Treatment Scale (ETS, German version) using a 4-point Likert scale with options of “partially disagree,” “partially agree,” “agree,” and “definitely agree.” Higher scores reflect a higher outcome expectation.

g Fear of Movement was assessed with the Tampa Scale for Kinesiophobia (TSK-GV, German version) using a 4-point Likert scale with options of “strongly disagree,” “disagree,” “agree,” and “strongly agree.” Higher scores reflect greater fear of movement.

### Primary Outcomes

**Figure 3.**
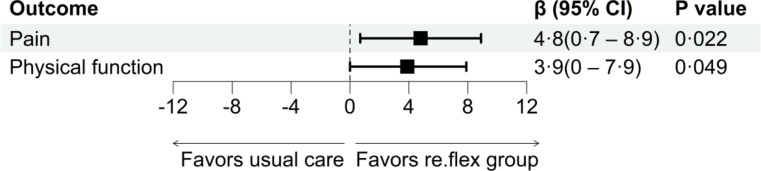
Primary outcomes pain and physical function.

The difference between study groups at 3 months was statistically significant for the primary outcome pain (β=4.8 points, 95% CI 0.7‐8.9; *P*=.02), with re.flex being superior to usual care. Cohen *d* was 0.35, demonstrating a small effect. After Bonferroni correction, no statistically significant difference was observed for physical function (β=3.9 points, 95% CI 0.0‐7.9; *P*=.049; Table 2, Figure 3, and Multimedia Appendix 1). The findings of the sensitivity analyses for the primary outcome pain confirmed the superiority of re.flex, with the exception of the conservative approach LOCF, which achieved a *P* value that was slightly above the significance level (Multimedia Appendix 1).

**Table 2.** Primary and secondary outcomes (primary analysis)^a^.

Outcomes	Change within group (3 months minus baseline)		Difference in change between groups (3 months)		
	re.flex group (n=98), mean (SD)	Usual care group (n=96), mean (SD)	β (95% CI)	*P* value	Cohen *d*
Primary outcomes					
Pain (KOOS Pain)^b^^,^^e^	9.7 (15.9)	4.5 (14.0)	4.8 (0.7-8.9)	.02	0.35
Physical function (KOOS ADL)^c^^,^^e^	9.0 (16.2)	4.7 (11.5)	3.9 (0-7.9)	.049	0.31
Secondary outcomes					
Symptoms (KOOS Symptoms)^e^	9.6 (17.9)	5.0 (14.4)	4.1 (–0.2 to 8.5)	.06	0.28
Function in sport and recreation (KOOS Sport and Recreation)^e^	9.5 (18.7)	5.2 (16.9)	4.5 (–0.8 to 9.7)	.09	0.24
Knee-related quality of life (KOOS QoL)^e^^,d^	4.1 (15.6)	1.6 (13.5)	2.4 (–1.6 to 6.5)	.24	0.18
Patients Global Assessment^f^^,^^g^	–0.1 (0.8)	–0.1 (0.7)	–0.1 (–0.3 to 0.1)	.36	0.10
VR-12 PCS^e^^,^^h^	1.7 (8.6)	–0.4 (6.6)	2.4 (0.3-4.5)	.03	0.28
VR-12 MCS^e^^,h^	–0.6 (10.2)	–0.3 (7.8)	–0.1 (–2.6 to 2.5)	.96	0.03
30s Chair Stand Test^e^	1.7 (1.9)	1.4 (1.9)	0.3 (–0.3 to 0.9)	.35	0.15

a Data are mean (SD). Differences in change between groups are adjusted for baseline and stratification variables.

b KOOS: Knee Osteoarthritis Outcome Score (0-100, worse to best).

c ADL: activities of daily living.

d QoL: quality of life.

e For change within groups, positive changes indicate improvement. For differences in change between groups, positive differences favor intervention group (re.flex).

f Patients' Global Assessment is scored 1-5 (best to worse).

g For change within groups, negative changes indicate improvement. For differences in change between groups, negative differences favor intervention group (re.flex).

h VR-12: Veterans RAND 12-Item Health Survey; PCS: Physical Component Score; MCS: Mental Component Score; Health-related quality of life (0-100, worse to best, normalized to a mean value of 50 (SD 10).

### Secondary Outcomes

For secondary outcomes, a statistically significant effect between study arms with superiority of re.flex was only observed for the PCS (β=2.4 points, 95% CI 0.3‐4.5; *P*=.03; Cohen *d*=0.28; Table 2 and Multimedia Appendix 1). Again, findings were confirmed by the sensitivity analyses (Multimedia Appendix 1).

### Subgroup Analyses

Regarding the subgroup analyses, the only significant interaction with the study arm (*P*=.03) was found for physical function in patients undergoing concomitant physiotherapy compared to patients not undergoing physiotherapy. Patients in the usual care group who received concomitant physiotherapy showed greater improvements than those with physiotherapy in the re.flex group. Conversely, patients in the re.flex group who did not receive concomitant physiotherapy demonstrated greater improvements than those of the usual care group. For all other subgroup variables, no significant interaction was found. Details on the number of patients receiving concomitant care and forest plots of subgroup analyses for the primary outcomes of pain and physical function can be found in the Multimedia Appendix 1.

### Responder Analyses

Details of the responder analyses can be found in Table 3. For each of the 3 transition scales (overall, pain, and function), positive effects (odds ratios and SRs >1) with significant benefits for the re.flex group could be found in the primary analysis (*P*<.001). The NNTs were between 2.6 and 3.4, and the SRs were between 2.1 and 2.7.

**Table 3.** Responder analyses for primary analysis.

Transition scales	re.flex group (3 months)^a^, n (%)	Usual care group (3 months)^a^, n (%)	OR (95% CI)^b^	*P* value	SR^c^	NNT^d^
Overall^e^	60 (61.2)	22 (22.9)	5.7 (2.9-11.0)	<.001	2.7	2.6
Pain^e^	56 (57.1)	26 (27.1)	3.8 (2.0-7.2)	<.001	2.1	3.3
Function^e^	53 (54.1)	24 (25)	3.8 (2.0-7.1)	<.001	2.2	3.4

a Only numbers for the category improved (somewhat better or much better).

b OR: odds ratio.

c SR: success ratio; quotient percentage of success in the re.flex group divided by percentage in the usual care group (“relative risk”).

d NNT: Number needed to treat.

e Subjective change in health status along the study period overall, for pain and walking (reflecting function) compared to 3 months ago was assessed using a 5-point Likert scale with options “much better,” “somewhat better,” “unchanged,” “somewhat worse,” “much worse”. Response scales were dichotomized into improved (somewhat better or much better) and not improved (unchanged, somewhat worse, or much worse).

### Safety, Adherence, Usability, and Satisfaction

AEs in the re.flex group were minor and mainly of a musculoskeletal nature. Overall, 12 AEs were sure (n=5), likely (n=5), or possibly (n=2) linked to the re.flex intervention. Thereof, 7 AEs led to the discontinuation of the training, and 4 AEs were needed to seek medical care (Multimedia Appendix 1). No intervention-related serious adverse events were reported. A more detailed analysis of exercise-related pain can be found in the Multimedia Appendix 1. The objectively measured overall exercise session adherence was 77% (2705/3528), indicating that 23% (823/3528) of all exercise sessions were not performed. This also includes sessions of participants who stopped the intervention before the end of the study. The attrition rate in the re.flex group increased during the study period from 2% (2/98) at week 1 to 28% (27/98) at week 12. Participants of the re.flex group reported a high level of usability and satisfaction with the re.flex app and a high treatment satisfaction. More details can be found in Table 4 and the Multimedia Appendix 1.

**Table 4.** Log files and usability of the re.flex app^a^.

Outcomes	re.flex group (n=98)	re.flex group (n=86)
Exercise session adherence (%)^b^^,c^	77	—^d^
Exercise repetition adherence (%), mean (SD)^c,e^	74 (43)	—
Active time (minutes), mean (SD)^f,g^	18 (6)	—
Usability (MAUQ)^h^ mean (SD)	—	5.4 (1.0)

a Missing data were not replaced with multiple imputation.

b Exercise session adherence is the percentage of conducted exercise sessions relative to the overall number of prescribed exercise sessions during the study period.

c Data refer to all patients of the re.flex group (n=98); 12 weeks with 3 exercise sessions per week.

d Not applicable.

e Exercise repetition adherence is the percentage of all valid repetitions in percent with a maximum value of 100%.

f Active time is the crude time for exercising with the sensor-equipped leg without login, calibration, video watching, and feedback on pain and exertion.

g Data refer to 2706 exercise sessions.

h MAUQ: mHealth App Usability Questionnaire (1-7, higher values indicating higher usability of the app).

## Discussion

### Principal Findings

This randomized controlled trial was designed to investigate patient benefit of the digital health app re.flex, which was designed as a self-directed intervention to instruct and guide exercises for patients with knee OA. Compared with usual care, re.flex showed superior findings with a small treatment effect in the primary outcome of pain at 3 months. No statistically significant difference was found for the primary outcome of physical function after adjustment for multiple testing. Except for the PCS score in the VR-12 instrument, no other secondary study outcomes did reveal significant treatment effects in favor of re.flex at 3 months. Overall, the study intervention showed a high adherence rate, high usability, and satisfaction with the re.flex app, and no intervention-related serious adverse events.

The findings and superiority of re.flex over usual care for the primary outcome of pain are in accordance with recent meta-analyses on the effectiveness of technology-based exercise interventions in patients with knee OA [20,21]. The meta-analyses revealed beneficial effects of using smartphone apps or web-based programs in reducing pain intensity [20,21]. In terms of improvements in physical function, the evidence is divergent, with studies reporting either no significant effect or only significant improvements when using the delivery-type app [20,21]. As only 5 [20] and 4 [21] studies with the delivery type app or web-based could be included, the quality of evidence is still low [35]. In addition, a direct comparison of study results is made difficult by the heterogeneity of study interventions, the presence or absence of human interaction, and sample characteristics. At present, nondigital interactions are the gold standard for exercise guidance, and the effectiveness of mHealth interventions should be comparable to this. Nondigital exercise interventions, delivered via one-to-one supervision, class-based programs, or home-based exercises, reveal small to medium effects for pain reduction and improvement in physical function [2,36]. Looking at the latest results of an individual participant data (IPD) meta-analysis on exercise therapy in knee and hip OA (short-term treatment effects of −6.4 points for pain and −4.5 for physical function; scale: 0‐100, best to worst) [37], the between-group differences of our study are quite comparable. However, the clinical relevance of the between-group differences in terms of pain and physical function has previously been questioned in the IPD meta-analysis. Thus, we would now like to situate our results according to these findings. In this context, we refer to our responder analysis, in which we assessed the subjective changes in OA-related health status of the participants and calculated the SR (“relative risk”) for treatment response. It was shown (see Table 3) that the risk for subjective improvement is between 2.1- and 2.7-fold higher in the re.flex group than in usual care. NNTs between 2.6 and 3.4 revealed smaller numbers than those reported in the latest Cochrane Review on land-based exercise therapy in knee OA derived from continuous variables (self-reported pain and physical function score on a scale of 0‐100, best to worst) [2], indicating a clinically relevant effect of the re.flex intervention. This is of particular importance as it also bears a societal value. Exercise is recommended as a first-line treatment in patients with knee OA [38,39], and digital health apps could address the undersupply of current health care treatment options. Moreover, the sensitivity analyses “MI ITT,” “complete case,” and “per protocol” showed similar results as the primary analysis for pain and physical function, indicating that the findings are robust (data are shown in the Multimedia Appendix 1). Despite the aforementioned findings, re.flex was not permanently listed in the DiGA directory in Germany, as the clinical relevance was questioned by the decision-making committee. As this is a recurring issue, for example, also addressed in the IPD meta-analysis by Holden and colleagues [37], we propose the development of precise criteria for clinical relevance in conservative OA therapy in the future.

The objective measure of the 30-Second Chair Stand Test failed to demonstrate any significant between-group differences. The within-group differences for both groups were found to be below the previously described improvements of clinical relevance of 2.5 repetitions in a population with knee OA receiving physiotherapeutic treatment [40]. Two reasons can explain the lack of improvement. One possibility is that the exercise intensity was insufficient to elicit adaptations in muscle gains. The values for perceived exertion recorded during the exercise sessions indicated a fairly moderate to somewhat strenuous intensity level. An efficacious training load would be considered to be within a strenuous to very strenuous range. In the future, it must be ensured that patients are well educated and supported in independently adjusting their exercise intensity. Another reason may be related to the construct validity and responsiveness of the 30-Second Chair Stand Test, as well as the correlations between the 30-Second Chair Stand Test and the PROMs pain and physical function that are considered to be low in the population of knee OA receiving conservative treatment. Therefore, improvements in pain and physical function may not be reflected in comparable improvements in the performance test, and the test should be used with caution to assess changes in functionality over time [41].

Adherence to exercise therapy is known to be essential for the success of treatment and the associated improvements in pain and physical function. Although supervised interventions are considered favorable for adherence to exercise [42], the use of digital apps for exercise guidance can also increase adherence level, as they enhance motivation and offer the opportunity to exercise independent of time and location. The special group of sensor-based apps can further increase adherence rates, as they give additional information and instruction on training execution and training load (eg, frequency and duration) and provide real-time biofeedback in order to prevent incorrect movement execution and teach correct movement patterns. So far, most digital-based exercise interventions were not supported by sensor technology. To our knowledge, only Mecklenburg and colleagues [11] also used a sensor-guided exercise approach and reported an adherence rate of 76% among those who started the program, which is on the same level as in our study (adherence to exercise 77%). Moreover, the adherence rate exceeds reported rates of 62%‐75% for nondigital home exercise interventions [43,44]. Ultimately, the occurrence of AEs and the dissatisfaction with treatment results observed in some patients also suggest that self-directed mHealth-based exercise training may not be the optimal choice for all individuals. This finding strengthens the conclusions of qualitative studies on digital exercise interventions for patients with knee OA, which have previously demonstrated that some patients require at least a certain degree of human supervision [22,45,46]. In this context, hybrid formats could be used to a greater extent.

### Strengths and Limitations

In general, the usage of usual care as the control group offers certain advantages and disadvantages. It enables the evaluation of an intervention’s effectiveness in relation to real-world conditions that are accessible to a wide patient population, thereby enhancing the generalizability of the results. Conversely, usual care can vary extensively across patients and health care systems and may induce bias due to its lack of standardization and replicability. According to the results of the subgroup analysis, we assume that concomitant physiotherapy did not positively influence the effectiveness of the study intervention. Further strengths of the trial are the large sample size, few missing outcome data (<10%), and a rigorous statistical analysis, including jump to reference for MI, as well as different sensitivity analyses that underpin the findings of the primary analysis. The risk of randomization bias was minimized, resulting in well-balanced baseline characteristics between study groups. The concomitant treatments during the interventional period were comparable for the most part, resulting in a low risk of bias related to deviations from intended interventions. A risk of bias in selection of the reported results can be ruled out, as the analysis was conducted according to the prespecified published study protocol. Another strength of the study is the use of motion sensors to collect accurate data on adherence to exercise. We can further assume a good external validity of the trial findings, as baseline characteristics (age, sex, and BMI) and baseline outcome measures for pain and physical function are in line with those reported in a recently published IPD meta-analysis on 31 studies [37] with more than 4240 participants affected by knee or hip OA. Therefore, findings should be generalizable to other patients with OA with similar disease severity in predominantly industrialized countries.

The trial has some limitations. As eligibility required a pain score of <60 (out of 0‐100, worst to best), findings may not be generalizable to individuals with milder symptoms. In addition, a selection bias toward sociodemographic characteristics cannot be ruled out, as baseline data demonstrate higher educational levels and only a small proportion of foreigners in comparison to the age-equal German population. Moreover, we have to state a risk of reporting bias as participants were not blinded and primary outcomes were self-reported. We must further acknowledge that using an MCID derived from minimal important improvements of within-group changes to evaluate between-group differences was inadequate. However, the between-group differences of a large IPD meta-analysis were of a similar size (3.8-6.4 for short-term improvements in physical function and pain) and can therefore serve as a benchmark [37]. Finally, a potential source of error for the training by an incorrect use of the sensors and technical features could not be ruled out. However, this risk is rather low, as the system has been designed to alert users to malfunctions.

### Conclusions

In summary, the findings of this randomized controlled trial provide evidence for the superiority of a self-directed sensor-based mHealth exercise intervention in comparison to usual care. The usage of the digitally delivered exercise program is associated with clinically meaningful improvements in knee-related pain in patients with knee OA. The high adherence rate, positive usability ratings, and high satisfaction levels with the app demonstrate that the implementation of a digital, self-directed exercise program is well accepted by the majority of users. However, minor exercise- and app-related AEs and dissatisfaction in some patients also illustrate that self-directed interventions are useful in many but not all patients with knee OA, and personal context factors should be considered when choosing the best delivery mode for exercises. In summary, a low-threshold and widely accessible treatment option, such as re.flex, can improve treatment access and effectiveness among patients with OA.

In the future, further research should evaluate supervised versus self-directed digital exercise delivery in knee OA and examine if the inclusion of sensors has an additional effect on movement quality or effectiveness. In addition, it would be important to focus on the development of selection criteria that allow accurate allocation of patients who may benefit from digital, self-directed exercise interventions and those who require more human support or are even better suited to traditional treatment.

## Supplementary material

10.2196/71558 Multimedia Appendix 1 Additional information.

10.2196/71558 Checklist 1 CONSORT-EHEALTH (V 1.6.1) checklist.

## Abbreviations

AE: adverse event
ANCOVA: analyses of covariance
CONSORT-EHEALTH: Consolidated Standards of Reporting Trials of Electronic and Mobile Health Applications and Online Telehealth
DiGA: Digitale Gesundheitsanwendung
DVG: Digitale-Versorgung-Gesetz
EHIS: European Health Interview Survey
GEDA: Gesundheit in Deutschland aktuell
HrQoL: health-related quality of life
IPD: individual participant data
ITT: intention-to-treat
KOOS: Knee Osteoarthritis Outcome Score
LOCF: last observation carried forward
MAUQ: mHealth App Usability Questionnaire
MCID: minimal clinically important difference
MCS: mental component score
mHealth: mobile health
MI: multiple imputation
NNT: numbers needed to treat
OA: osteoarthritis
PCS: physical component score
PGA: patient global assessment
PROM: patient-reported outcome measure
QoL: quality of life
SR: success ratio
VR-12: Veterans RAND 12-Item Health Survey
