# Supplementary material for: Effectiveness of the Self-Directed mHealth Exercise Intervention re.flex in Patients With Knee Osteoarthritis: Randomized Controlled Trial
Source: J Med Internet Res. 2025 Oct 9;27:e71558. doi: 10.2196/71558 (PMC12510439; doi:10.2196/71558)
Supplement: Multimedia Appendix 1 [file jmir-v27-e71558-s001.docx]

**Appendix 1 to „Effectiveness of the self-directed m-Health exercise intervention re.flex in patients with knee osteoarthritis: A randomized controlled trial**.”

This appendix has been provided by the authors to give readers additional information about the work.

Table of Contents

Development process of the intervention 2

Table A1: 12-week exercise program (re.flex) for patients with knee osteoarthritis – phases, dosage principles and objectives. 3

Table A2: Exercise program. 4

App features and course of a training session 5

Table A3: Outcome measures and study instruments. 12

Sample size 14

Table A4: Baseline characteristics of participants (incl. comparison between study groups). 15

Table A5: Mean (SD) scores on primary and secondary outcome measures for baseline (t0) and after three months (t1), by study group. 17

Table A6: Sensitivity analyses for primary and secondary outcomes. 18

Subgroup analyses 20

Adverse Events 23

Exercise-related pain 24

Table A10: Logfile data for week 1, week 12, and overall. 25

Table A11: Perceived exertion and pain outcomes before, during, and after exercising. 25

Table A12: Patient satisfaction of the re.flex app and treatment satisfaction. 25

References 26

# Development process of the intervention

Kineto Tech Rehab SRL^©^ (Romania) developed the exercise app including features for visual and verbal feedback, monitoring, push-notifications, chat function for technical issues and two sensors to allow biofeedback on knee joint motion. The app was then optimized by implementing a graded 12-week exercise program specifically designed for the patient target group according to disease-specific recommendations (1-3) and the experience of the study team in planning and conducting exercise interventions for patients with hip and knee OA (4-7). In addition, exercise videos were optimized, the range of motion for each exercise defined to allow proper biofeedback on movement control, and app features such as monitoring of perceived exertion and pain have been added.

Re.flex underwent repeated usability testing and was further evaluated in a pilot study (8). At the beginning of the evaluation period, the software was only available for iOS devices. Starting on Mar 06, 2023, it was expanded for use on Android operating systems. This study evaluated the latest version of re.flex (iOS 1.1.81 and Android 1.0.10) at time of intervention completion of the intervention group. Minor bug fixes were made concerning app design and technical issues, e.g. device crashes. No new features were implemented and functional components of the app (e.g. the exercise program) were not changed during the study phase.

# Table A1: 12-week exercise program (re.flex) for patients with knee osteoarthritis – phases, dosage principles and objectives.

| **Phase** | **Week** | **General information** | **Sets and repetitions/seconds by training method** | **Objectives** |
| --- | --- | --- | --- | --- |
| 1 | 1-2 | - - - - 3 sessions à 5 exercises per week       - Each session lasting 25-30 minutes       - Training material: chair, ball or pillow, elastic resistance bands | - Strengthening exercises: 2x25 repetitions - Balance tasks: 6x15 seconds (bilateral exercises), 3x15 seconds on each leg (unilateral exercises) - Mobilization exercises: 1x30 repetitions - Stretching exercises: 2x20 seconds - All one-sided exercises were conducted alternately | - Familiarization with different kinds of exercises and exercise loads - Get to know self-determined adaption of exercise intensity to the perceived load and pain - Possibility to choose between two different intensity levels for strengthening exercises |
| 2 | 3-6 |  |  | - Increase strength endurance - Enhance range of motion of the lower extremities - Improve balance ability - Possibility to choose between two different intensity levels for strengthening exercises |
| 3 | 7-12 |  | - Strengthening exercises: 3x15 repetitions - Balance tasks: 6x15 seconds (bilateral exercises), 3x15 seconds on each leg (unilateral exercises) - Mobilization exercises: 1x30 repetitions - Stretching exercises: 2x20 seconds - All one-sided exercises were conducted alternately | - Muscle building with higher intensities and lower repetition numbers - Becoming familiar with further exercise variations with higher intensities - Further improvement of balance and range of motion - Increased ability to independently select and adapt exercise difficulty and load dosage depending on the perception of exertion and pain - Possibility to choose between two different intensity levels for strengthening exercises |

# Table A2: Exercise program.

| **App-based home exercise program**   - 3 sessions à 5 exercises per week - Each session lasting 25-30 minutes - Training material: chair, ball or pillow, elastic resistance bands (provided by the study) - Primary focus was to strengthen knee extensors, knee flexors and hip abductors; further exercises comprised balance, mobilization and stretching - Patients were asked to conduct strengthening exercises with an intensity of 7-9 (“exhausting to very exhausting” on a scale 0-10) - Balance tasks should be challenging, but always able to be performed correctly | | | |
| --- | --- | --- | --- |
| 1. Strengthening exercises | | | |
| Knee extension | Supine | S1. Knee extension | **Progression:** without ground contact |
|  | Seated | S2. Knee extension | **Progression:** without ground contact |
|  |  | S3. Resistive knee extension | **Progression:** without ground contact |
|  | Standing | S4. Knee extension with band | **Progression:** without ground contact, increase resistance with elastic band |
| Knee flexion | Standing | S5. Knee flexion | **Progression:** without ground contact |
|  |  | S6. Knee flexion with band | **Progression:** without ground contact, increase resistance with elastic band |
|  |  | S7. Resistive knee flexion | **Progression:** without ground contact |
| Hip abduction | Seated | S8. Hip abduction | **Progression:** with elastic band, increase resistance with elastic band |
|  | Standing | S9. Hip abduction with band | **Progression:** without ground contact, increase resistance with elastic band |
| Hip flexion | Supine | S10. Hip flexion | **Progression:** without ground contact |
|  |  | S11. Lift stretched leg | **Progression:** without ground contact |
|  | Seated | S12. Hip flexion | **Progression:** without ground contact |
|  |  | S13. Hip flexion with extended leg | **Progression:** without ground contact |
|  | Standing | S14. Hip flexion without band | **Progression:** without ground contact |
|  |  | S15. Hip flexion with band | **Progression:** without ground contact, increase resistance with elastic band |
| Hip extension | Standing | S16. Hip extension without band | **Progression:** without ground contact |
|  |  | S17. Hip extension with band | **Progression:** without ground contact, increase resistance with elastic band |
| Sit-to-stand | Seated | S18. Getting up from the seat | **Progression:** without arm support |
|  |  | S19. Getting up from the seat with band | **Progression:** without arm support, increase resistance with elastic band |
|  |  | S20. Single legged getting up from the seat | **Progression:** without arm support |
| Squat variation | Standing | S21. Mini squat | **Progression:** upper body vertical |
|  |  | S22. Mini squat single-legged | **Progression:** upper body vertical |
|  |  | S23. Wall slide | **Progression:** upper body vertical |
|  |  | S24. Wall slide with band | **Progression:** upper body vertical, increase resistance with elastic band |
|  |  | S25. Wall slide with ball | **Progression:** upper body vertical |
|  |  | S26. Single leg wall slide | **Progression:** upper body vertical |
|  |  | S27. Stairs upwards | **Progression:** without ground contact |
|  |  | S28. Stairs downwards | **Progression:** without ground contact |
| 1. Balance exercises | | | |
| Standing | | B1. Double legged standing stable | **Progression:** eyes closed |
|  |  | B2. Double legged standing stable with destabilization of the arms |  |
|  |  | B3. Tandem standing stable | **Progression:** eyes closed |
|  |  | B4. Tandem standing with destabilization of the arms |  |
|  |  | B5. Single-leg standing stable | **Progression:** eyes closed |
|  |  | B6. Step position with weight shift to the front |  |
|  |  | B7. Step position with weight shift to the back |  |
| 1. Mobilization exercises | | | |
| Supine | | M1. Knee extension/flexion |  |
| Seated | | M2. Knee extension/flexion |  |
|  |  | M3. Patella relief |  |
| 1. Stretching exercises | | | |
| Seated | | St1. Stretch musculature of the thigh back (ischiocrural musculature) |  |
| Standing | | St2. Stretch musculature of the thigh back (ischiocrural musculature) |  |
|  |  | St3. Stretching the front of the thigh |  |

# App features and course of a training session

1. Home screen

| 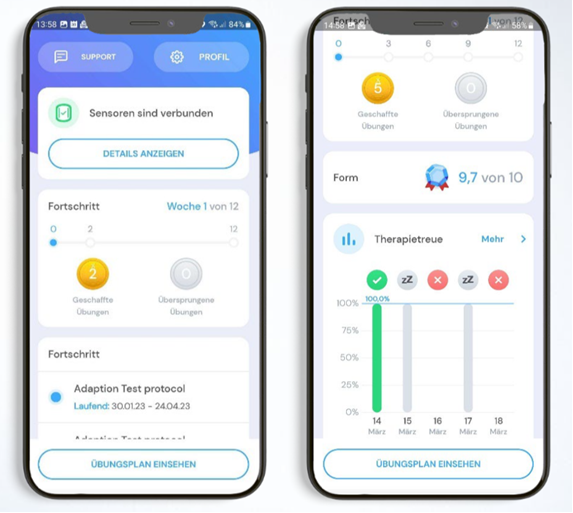 |
| --- |
| **Figure A1: Home screen after login.** |

1. Overview training schedule

| 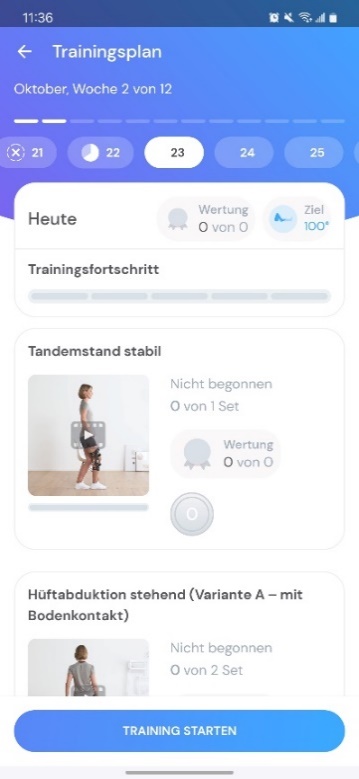 | 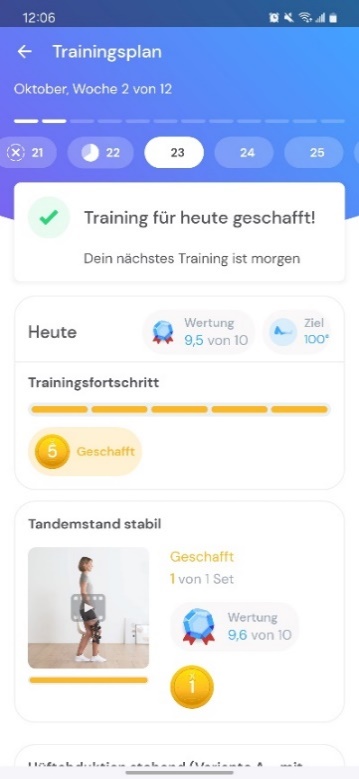 |
| --- | --- |
| **Figure A2: Daily training schedule with overview of scheduled exercises.** | **Figure A3: Daily training schedule after completing the exercises.** |

1. Process of connecting and calibrating sensors

| 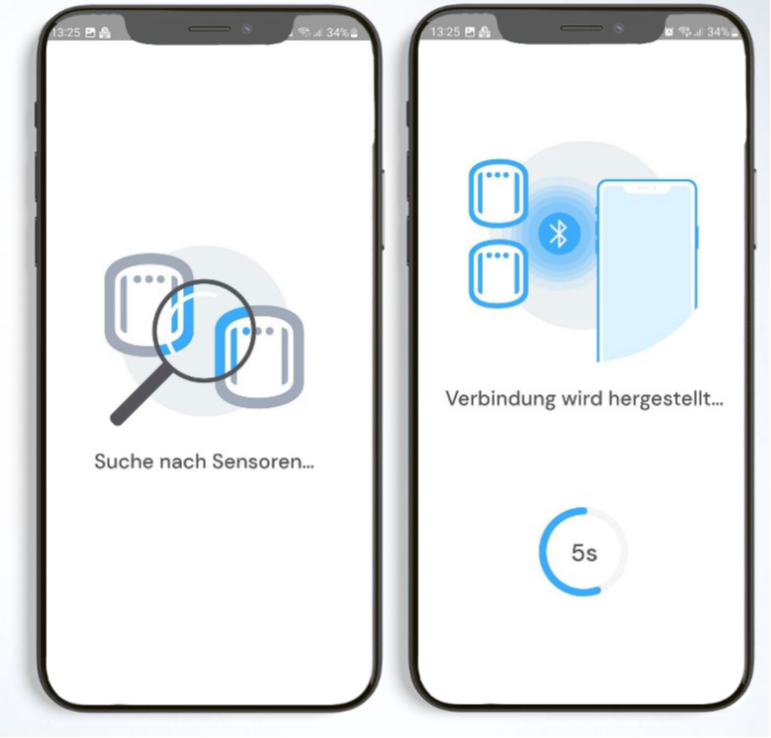 |
| --- |
| **Figure A4: Connecting the sensors** |
| 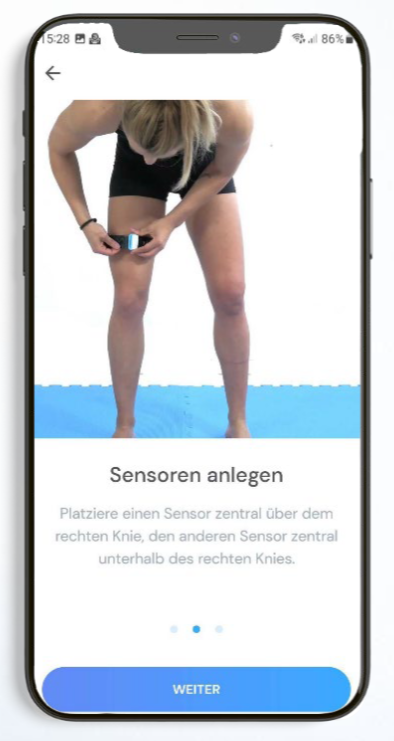 |
| **Figure A5: Attaching sensors proximally and distally to the osteoarthritis-affected knee joint.** |

| 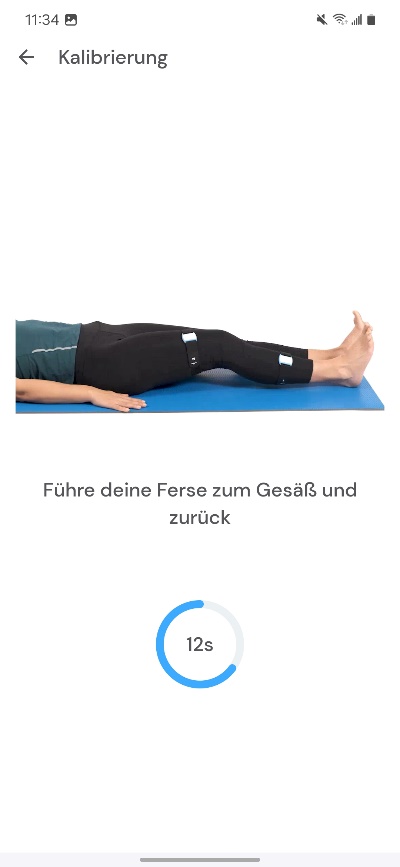 | 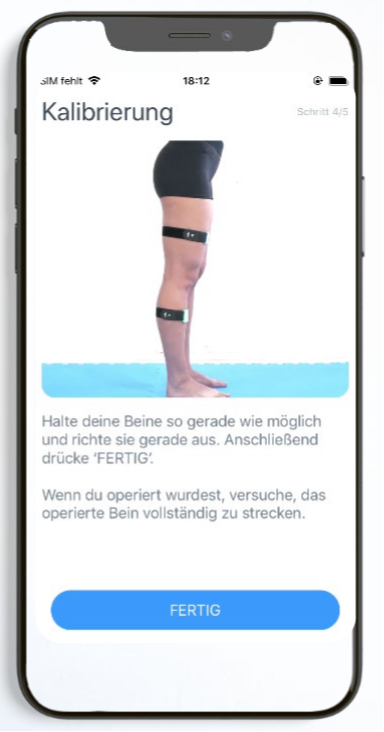 |
| --- | --- |
| **Figure A6: Movement task during calibration in supine position.** | **Figure A7: Movement task during calibration in standing position.** |

1. Training process
   1. Exercise description and video

| 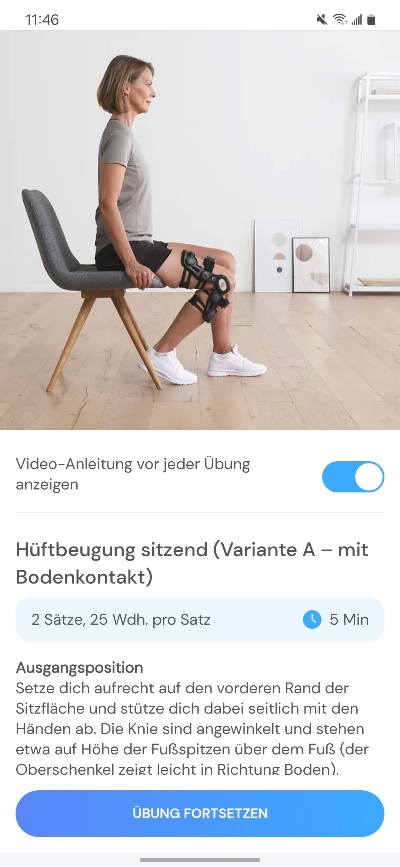 | 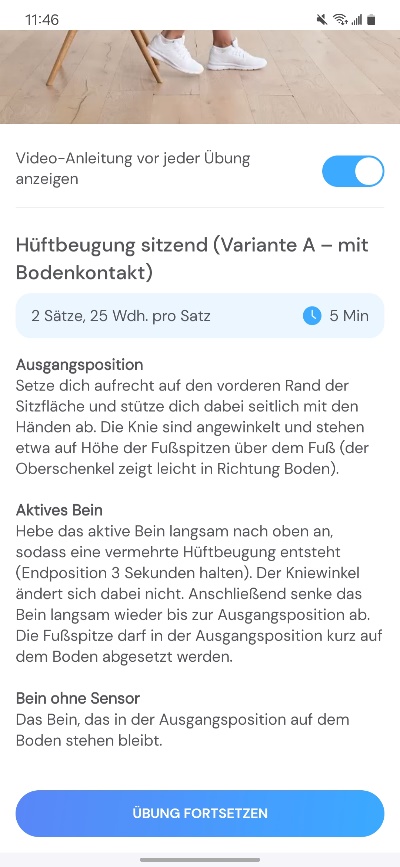 |
| --- | --- |
| **Figure A8: Video for the exercise execution.** | **Figure A9: Text description for the exercise execution.** |

- 1. Avatar

| 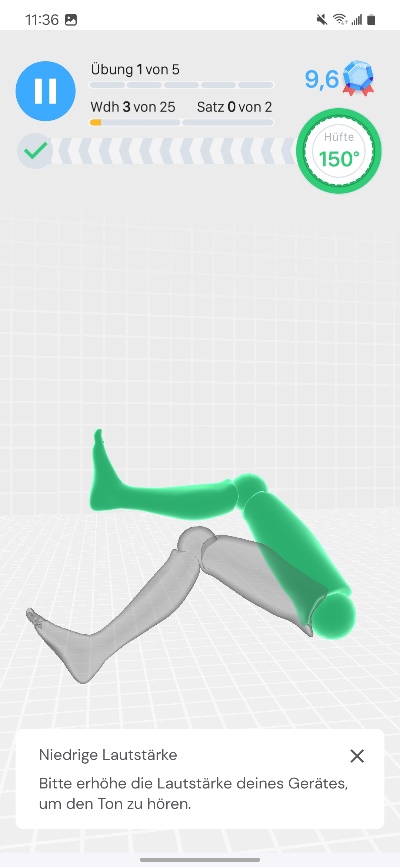 |
| --- |
| **Figure A10: The patient must align his virtual limb (green) to the target condition displayed with another avatar (grey) regarding pre-defined range of motion, movement velocity, and number of repetitions.** |

- 1. Movement bar

| 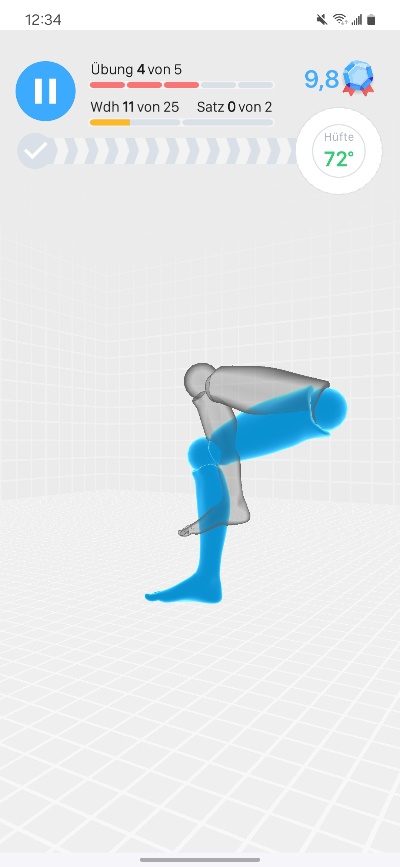 | 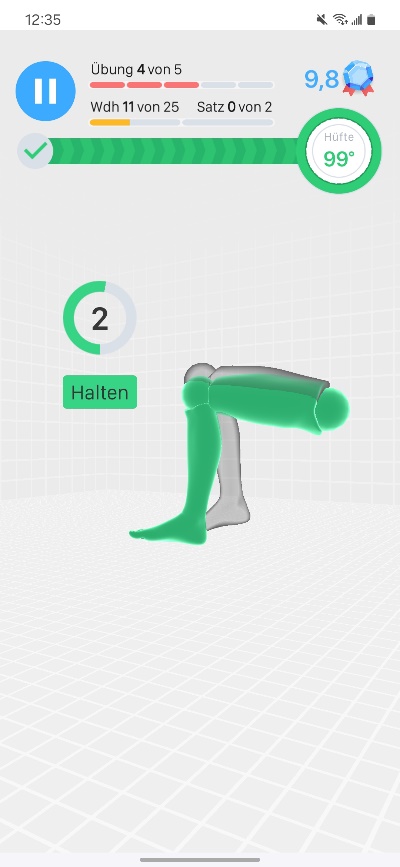 |
| --- | --- |
| **Figure A11: The own training leg is blue in the starting position. The movement bar with the target angle is displayed on the top.** | **Figure A12: The own training leg and the movement bar turn green when the target position is reached.** |

- 1. Repetition counting

| 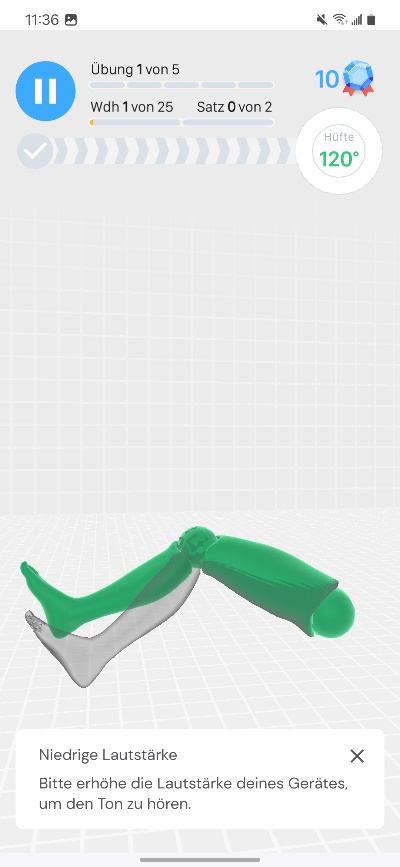 |
| --- |
| **Figure A13: Repetitions and the number of sets are displayed above the movement bar and registered when performed correctly.** |

- 1. Correction of exercise execution

| 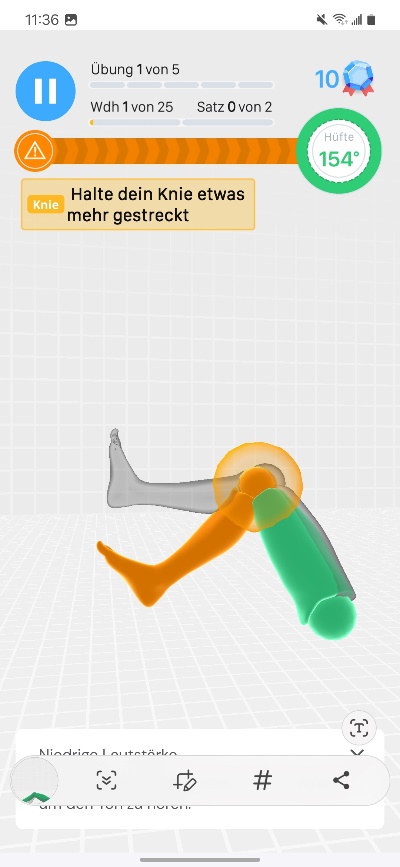 | 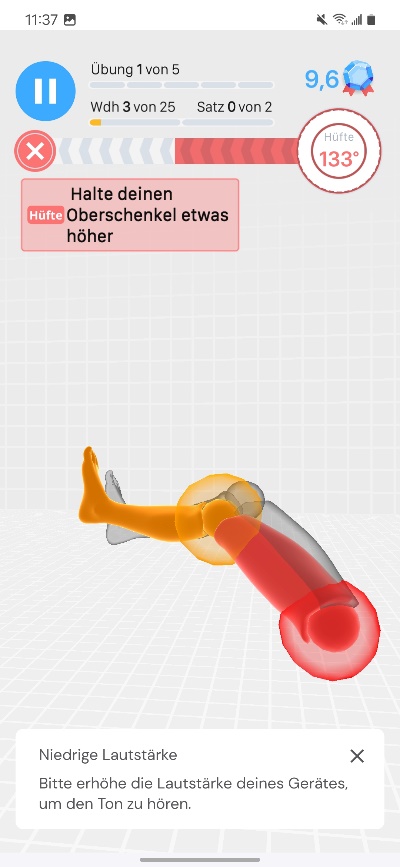 |
| --- | --- |
| **Figure A14: Advice: Knee should be extended more.** | **Figure A15: Advice: Thigh should be hold in a higher position.** |

- 1. Perceived pain and strain

| 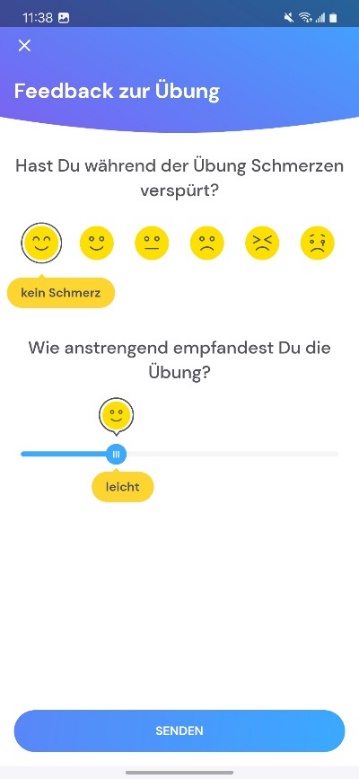 | 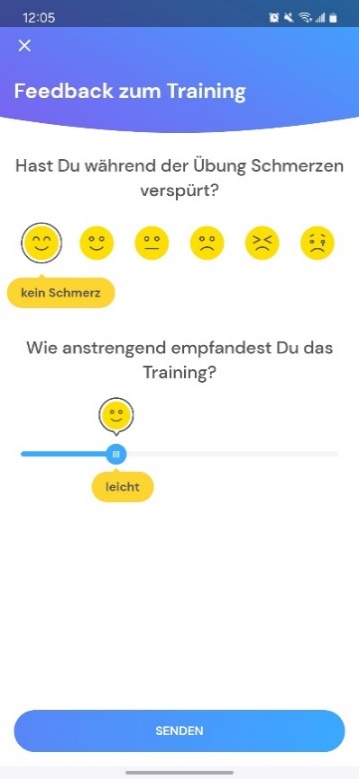 |
| --- | --- |
| **Figure A16: Pain and strain query after each exercise.** | **Figure A17: Pain and strain query after the exercise session.** |

- 1. Change exercise variation or skip exercise

| **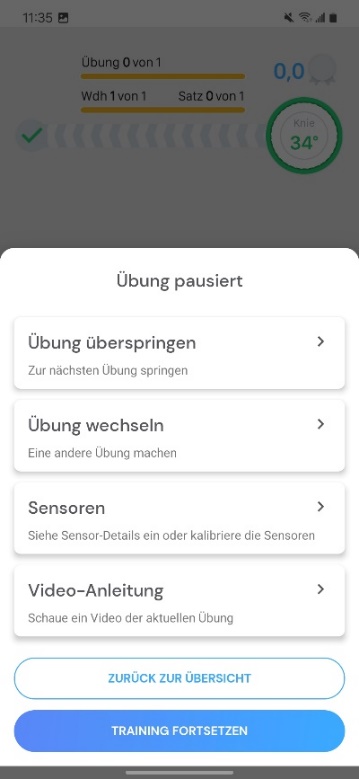** | 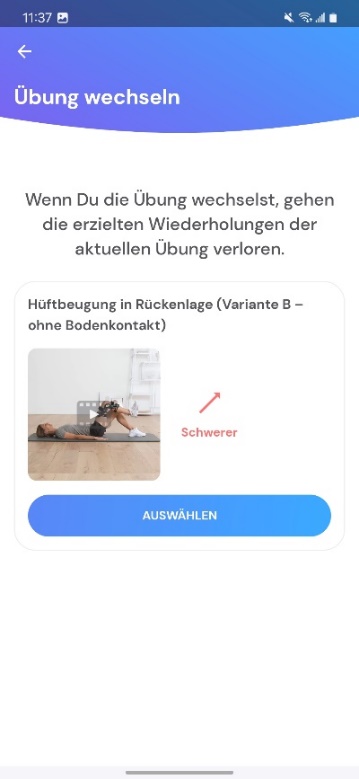 |
| --- | --- |
| **Figure A18: Exercises can be paused or skipped.** | **Figure A19: Users can choose one of two difficulty levels for strengthening exercises.** |

1. Monitoring / Statistics

| 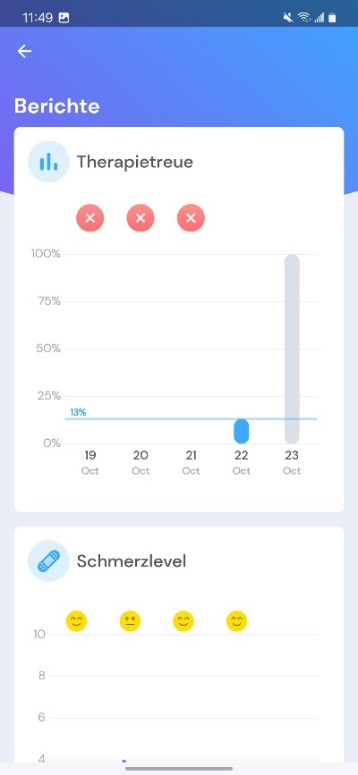 | 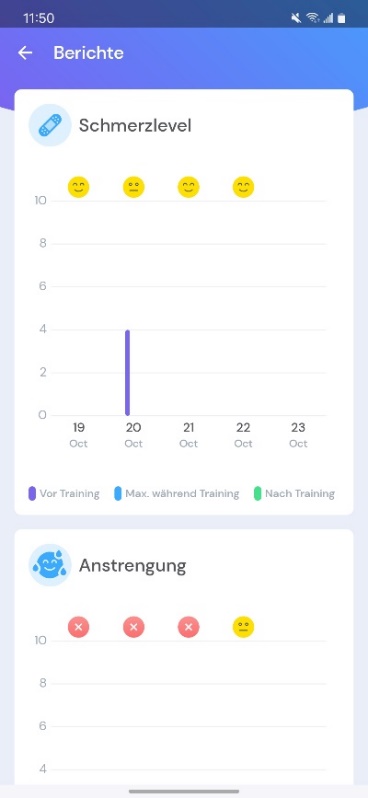 | 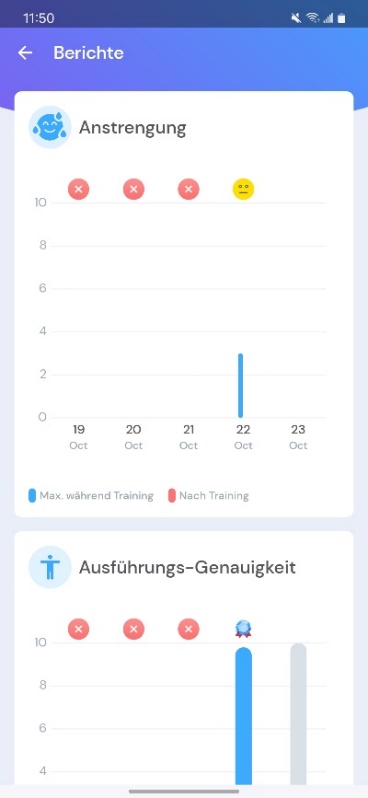 |
| --- | --- | --- |
| **Figure A20: Monitoring of exercise adherence.** | **Figure A21: Monitoring of perceived pain level.** | **Figure A22: Monitoring of perceived exertion.** |

1. In-App Chat support

| 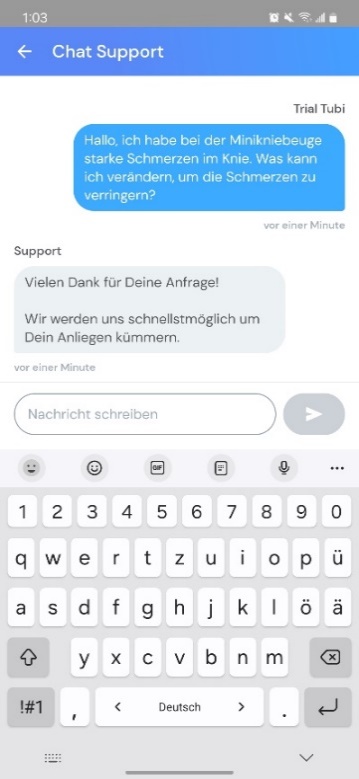 |
| --- |
| **Figure A23: In-App Chat support.** |

# Table A3: Outcome measures and study instruments.^a^

| **Outcome Description** | **Instrument [ref]** | **Scale & Score** | **Items** | **Sample** | **Timepoints** |
| --- | --- | --- | --- | --- | --- |
| Socio-demographic data, anthropometric data, other baseline data | Variables and definitions according to the International Standard Set of Outcome measures for patients with hip or knee OA (9). | NA | NA | IG, CG | t0 |
| Technical affinity towards electronic devices | Self-reported questionnaire on subjective technical affinity (Technical Affinity - Electronic Devices (TA-EG)) (10) | 5-point Likert (b2w), -2-14 (w2b) | 17 | IG, CG | t0 |
| Outcome expectation | Expectation for Treatment Scale (ETS, German version) (11) | 4-point Likert (w2b), 5-20 (w2b) | 5 | IG, CG | t0 |
| Fear of Movement | Tampa Scale for Kinesiophobia (German Version, TSK-GV) (12) | 4-point Likert (w2b), 6-24 (b2w) | 6 | IG, CG | t0 |
| Subscale knee pain (Co-1° outcome) | Knee Osteoarthritis Outcome Score (KOOS) (13, 14): A disease related questionnaire asking for patient’s opinion about their knee complaints. | 5-point Likert (b2w), 0-100 (w2b) | 9 | IG, CG | t0, t1 |
| Subscale physical function (Activities in daily living, ADL) (Co-1° outcome) | Knee Osteoarthritis Outcome Score (KOOS) (13, 14): A disease related questionnaire asking for patient’s opinion about their knee complaints. | 5-point Likert (b2w), 0-100 (w2b) | 17 | IG, CG | t0, t1 |
| Subscales symptoms, function in sport and recreation (Sport/Rec), knee-related quality of life (QoL) | Knee Osteoarthritis Outcome Score (KOOS) (13, 14): A disease related questionnaire asking for patient’s opinion about their knee complaints. | 5-point Likert (b2w), 0-100 (w2b) | 7, 5, 4 | IG, CG | t0, t1 |
| Patient’s global assessment (PGA) | Patient Global Assessment of osteoarthritis – Knee (15) | 5-point Likert (b2w) | 1 | IG, CG | t0, t1 |
| Health-related quality of life (HRQoL) – physical component score (PCS) | Veterans RAND 12-Item Health Survey (VR-12) (16, 17): A generic questionnaire to assess the patient’s opinion about their health-related quality of life (HRQoL). | 3-point, 5-point, or 6-point Likert, 0-100 (w2b) | 12 | IG, CG | t0, t1 |
| Health-related quality of life (HRQoL) – mental component score (MCS) | Veterans RAND 12-Item Health Survey (VR-12) (16, 17): A generic questionnaire to assess the patient’s opinion about their health-related quality of life (HRQoL). | 3-point, 5-point, or 6-point Likert, 0-100 (w2b) | 12 | IG, CG | t0, t1 |
| Subjective assessment of overall change, change in pain and change in function compared to three months ago | Transition question according to Angst, Benz (18) | 5-point Likert (b2w), dichotomized into improved (somewhat/much better) and not improved (unchanged, somewhat/much worse) | 3 | IG, CG | t1 |
| Functional strength measure for the lower extremities | 30-sec Chair Stand Test (19) | Number of repetitions | NA | IG, CG | t0, t1 |
| Treatment progression | Variables and definitions according to the International Standard Set of Outcome measures for patients with hip or knee OA (9). | NA | NA | IG, CG | t0, t1 |
| Care utilization |  | NA | NA | IG, CG | t0, t1 + after 4 and 8 weeks |
| Usability of the app | m-Health App Usability Questionnaire (MAUQ) (20) | 7-point Likert (w2b), 1-7 (w2b) | 18 | IG | t1 |
| Patient satisfaction with the app | Patient satisfaction questionnaire (ZUF-8) (21) | 4-point Likert, 8-32 (w2b) | 8 | IG | t1 |
| Patient satisfaction with the results of the treatment | Satisfaction with the results (9) | 5-point Likert (b2w) | 1 | IG, CG | t1 |
| Adherence to exercise | Logfiles relate to overall exercise session adherence (percentage of conducted exercise sessions relative to the overall number of prescribed exercise sessions) and exercise repetition adherence (percentage of all valid repetitions in percent with a maximum value of 100%). | NA | NA | IG | continuously during intervention phase |
| Active time | Logfiles: crude time for exercising with the sensor-equipped leg without login, calibration, video watching, and feedback on pain and exertion. | NA | NA | IG | continuously during intervention phase |

**Table A3: Outcome measures and study instruments (continued).**

| Rating of perceived exertion (RPE) during and after exercising | Entry into the app after each exercise and after the training using an adapted RPE-Scale (NRS 0-10). | 0-10 (no exertion to maximal exertion) | NA | IG | continuously during intervention phase |
| --- | --- | --- | --- | --- | --- |
| Rating of perceived pain   - Delta pain (rating of perceived pain after – before exercising) - Perceived pain during exercising | Entry into the app before/after the training and after each exercise using a faces pain scale referring to the numbers 0, 2, 4, 6, 8, 10. | 0-10 (no pain to highest pain) | NA | IG | continuously during intervention phase |
| Exercise-related pain | Retrospective questions concerning exercise-related pain (single exercise or whole exercise session) including frequency, duration, and intensity | NA | NA | IG, CG | t1 |
| Adverse event report | Direct contact to study personal | NA | NA | IG, CG | if reported |
| ^a^ b2w=best to worse. w2b=worse to best. IG=intervention group. CG=control group. t0=timepoint 0, collection point at baseline. t1=timepoint 1, collection point after three months. NRS=Numerical Rating Scale. | | | | | |

# Sample size

The following excerpt is taken from the published study protocol (22).

“The two primary endpoints of the study are the KOOS subscales of pain and ADL. The results of a pilot study comparing the KOOS pain subscale and KOOS ADL subscale of 29 patients in the control group (usual care) with 15 patients in the intervention group (re.flex) using baseline adjusted analysis of covariance revealed effect sizes of 1.16 (Pain subscale) and 1.03 (ADL subscale). A recent meta-analysis on exercise interventions in patients with knee OA reported a standardized mean difference of 0.5 (95% CI 0.37 to 0.63) for pain reduction immediately posttreatment in comparison to usual care or minimal treatment (23). Additionally, minimal clinically important differences (MCIDs) were reported between 5.5 and 8.7 points on the WOMAC pain subscale (score 0–100) for nonsurgical treatment strategies in patients with knee OA (24, 25). These correspond to standardized mean differences between 0.59 and 0.94. Based on the preceding, rather heterogeneous results with effect sizes between 0.5 and 1.2 from the results of the pilot study (8) and findings and recommendations of other sources, the planned study is powered to demonstrate a MCID of 5 points (0–100) on the KOOS Pain Subscale between the intervention and control group with a standard deviation of 10. This leads to an effect size of 0.5 and to a sample size of evaluable participants of 2*78=156. For adjustment of baseline, aetiology, medication and laterality, 4 additional degrees of freedom are spent, and the sample size is increased to 160. Considering a drop-out rate of ~ 20%, 200 patients will be recruited to achieve a power of 80% with a type 1 error of 0.025 (two-sided Bonferroni correction for two confirmatory outcomes) by baseline adjusted comparison of outcome values at t1 between study arms (analysis of covariance, ANCOVA).”

# Table A4: Baseline characteristics of participants (incl. comparison between study groups).^a^

|  | **Total** | **re.flex group (n=98)** | **Usual care group (n=96)** | ***P* value** |
| --- | --- | --- | --- | --- |
| **Demographics** | | | |  |
| Age, years | 61.9 (7.7) | 61.6 (8.0) | 62.1 (7.5) | .65^b^ |
| Gender |  |  |  | .31^c^ |
| Female | 132 (68%) | 70 (71%) | 62 (65%) | .. |
| Male | 62 (32%) | 28 (29%) | 34 (35%) | .. |
| **Anthropometry** |  |  |  |  |
| Body Height, cm | 168.9 (9.0) | 168.2 (8.8) | 169.5 (9.3) | .30^b^ |
| Body Weight, kg | 81.8 (72.0-93.4) | 82.2 (74.8-93.0) | 81.6 (69.0-93.7) | .37^d^ |
| Body-mass-index, kg/m² | 28.1 (25.9-31.9) | 28.4 (26.8-32.2) | 27.8 (25.0-31.4) | .08^d^ |
| **Stratification factors** | | | |  |
| Laterality |  |  |  | .97^c^ |
| One-sided knee OA | 73 (38%) | 37 (38%) | 36 (38%) | .. |
| Both-sided knee OA | 121 (62%) | 61 (62%) | 60 (62%) | .. |
| Etiology |  |  |  | .88^c^ |
| Primary knee OA | 167 (86%) | 84 (86%) | 83 (87%) | .. |
| Secondary knee OA | 27 (14%) | 14 (14%) | 13 (13%) | .. |
| Medication (knee OA-related) |  |  |  | .80^c^ |
| No or sporadic | 181 (93%) | 91 (93%) | 90 (94%) | .. |
| Regularly | 13 (7%) | 7 (7%) | 6 (6%) | .. |
| **Socio economics** | | | |  |
| Nationality |  |  |  | .98^c^ |
| German | 188 (97%) | 95 (97%) | 93 (97%) | .. |
| Others | 6 (3%) | 3 (3%) | 3 (3%) | .. |
| Life situation |  |  |  | .29^e^ |
| Working | 109 (56%) | 61 (62%) | 48 (50%) | .. |
| Unemployed | 1 (1%) | 0 (0%) | 1 (1%) | .. |
| Retired | 77 (40%) | 34 (35%) | 43 (45%) | .. |
| Others | 7 (4%) | 3 (3%) | 4 (4%) | .. |
| Education |  |  |  | .53^e^ |
| Low (<10 years) | 17 (9%) | 8 (8%) | 9 (9%) | .. |
| Middle (≥10 years) | 70 (36%) | 36 (37%) | 34 (35%) | .. |
| High (university entrance qualification) | 97 (50%) | 49 (50%) | 48 (50%) | .. |
| Others | 10 (5%) | 5 (5%) | 5 (5%) | .. |
| Job Qualification |  |  |  | .38^c^ |
| None | 3 (2%) | 1 (1%) | 2 (2%) | .. |
| Apprenticeship | 98 (51%) | 50 (51%) | 48 (50%) | .. |
| Administration | 5 (3%) | 4 (4%) | 1 (1%) | .. |
| University of appl. sciences | 22 (11%) | 12 (12%) | 10 (10%) | .. |
| University | 33 (17%) | 12 (12%) | 21 (22%) | .. |
| PhD | 10 (5%) | 7 (7%) | 3 (3%) | .. |
| Others | 23 (12%) | 12 (12%) | 11 (12%) | .. |
| Housing |  |  |  | .99^c^ |
| Together with partner | 165 (85%) | 83 (85%) | 82 (85%) | .. |
| Alone | 27 (14%) | 14 (14%) | 13 (14%) | .. |
| Others | 2 (1%) | 1 (1%) | 1 (1%) | .. |
| **Anamnesis** | | | |  |
| Time since diagnosis of knee osteoarthritis, months | 63 (24-120) | 65 (24-120) | 61 (24-120) | .85^d^ |
| Osteoarthritis |  |  |  | .. |
| Right knee | 159 (82%) | 80 (82%) | 79 (82%) | .91^c^ |
| Left knee | 154 (79%) | 79 (81%) | 75 (78%) | .67^c^ |
| Right hip | 3 (2%) | 1 (1%) | 2 (2%) | .55^c^ |
| Left hip | 6 (3%) | 5 (5%) | 1 (1%) | .10^c^ |
| Signal joint - Knee |  |  |  | .38^c^ |
| Right | 109 (56%) | 52 (53%) | 57 (59%) | .. |
| Left | 85 (44%) | 46 (47%) | 39 (41%) | .. |
| Difficulty/pain severity of the knee osteoarthritis^f^ |  |  |  | .73^c^ |
| No | 1 (1%) | 0 (0%) | 1 (1%) | .. |
| Mild | 52 (27%) | 27 (28%) | 25 (26%) | .. |
| Moderate | 123 (63%) | 61 (62%) | 62 (65%) | .. |
| Severe | 18 (9%) | 10 (10%) | 8 (8%) | .. |
| Extreme | 0 (0%) | 0 (0%) | 0 (0%) | .. |
| **History of injury/disease** | | | |  |
| Ligament injury | 43 (22%) | 22 (22%) | 21 (22%) | .92^c^ |
| Meniscus damage | 107 (55%) | 50 (51%) | 57 (59%) | .24^c^ |
| Trauma | 24 (12%) | 11 (11%) | 13 (14%) | .62^c^ |
| Developmental disorder | 8 (4%) | 4 (4%) | 4 (4%) | .98^c^ |
| Other joint disease | 7 (4%) | 4 (4%) | 3 (3%) | .72^c^ |
| No previous history | 56 (29%) | 31 (32%) | 25 (26%) | .39^c^ |
| **Prior Therapy** | | | |  |
| Anterior cruciate ligament (ACL) reconstruction right | 12 (6%) | 8 (8%) | 4 (4%) | .26^c^ |
| Anterior cruciate ligament (ACL) reconstruction left | 16 (8%) | 7 (7%) | 9 (10%) | .56^c^ |
| Meniscus operation right | 52 (27%) | 28 (29%) | 24 (25%) | .61^c^ |
| Meniscus operation left | 47 (24%) | 21 (21%) | 26 (27%) | .36^c^ |

**Table A4: Baseline characteristics of participants (incl. comparison between study groups) (continued).**

|  | **Total** | **re.flex group (n=98)** | **Usual care group (n=96)** | ***P* value** |
| --- | --- | --- | --- | --- |
| Osteotomy right | 6 (3%) | 3 (3%) | 3 (3%) | .97^c^ |
| Osteotomy left | 2 (1%) | 1 (1%) | 1 (1%) | .98^c^ |
| Joint replacement right | 4 (2%) | 1 (1%) | 3 (3%) | .29^c^ |
| Joint replacement left | 7 (4%) | 3 (3%) | 4 (4%) | .67^c^ |
| Number of total hip arthroplasty |  |  |  | .03^g^ |
| 0 | 183 (94%) | 89 (91%) | 94 (98%) | .. |
| 1 | 8 (4%) | 6 (6%) | 2 (2%) | .. |
| 2 | 3 (2%) | 3 (3%) | 0 (0%) | .. |
| No surgeries | 83 (43%) | 43 (44%) | 40 (42%) | .76^c^ |
| **Comorbidities** | | | |  |
| Heart disease | 14 (7%) | 6 (6%) | 8 (8%) | .58^c^ |
| Hypertension | 68 (36%) | 35 (37%) | 33 (34%) | .72^c^ |
| Stroke (burden) | 0 (0%) | 0 (0%) | 0 (0%) | .. |
| Circulatory disorder | 3 (2%) | 2 (2%) | 1 (1%) | .55^c^ |
| Lung | 12 (6%) | 7 (7%) | 5 (5%) | .55^c^ |
| Diabetes | 6 (3%) | 3 (3%) | 3 (3%) | 1.00^c^ |
| Kidney | 1 (1%) | 1 (1%) | 0 (0%) | .32^c^ |
| Neurological Disease | 1 (1%) | 0 (0%) | 1 (1%) | .32^c^ |
| Liver | 3 (2%) | 1 (1%) | 2 (2%) | .56^c^ |
| Cancer | 9 (5%) | 6 (6%) | 3 (3%) | .31^c^ |
| Depression | 8 (4%) | 4 (4%) | 4 (4%) | 1.00^c^ |
| Back | 22 (12%) | 14 (15%) | 8 (8%) | .17^c^ |
| **Activity and training variables** | | | |  |
| Physical activity (per week) |  |  |  | .82^c^ |
| No | 5 (3%) | 3 (3%) | 2 (2%) | .. |
| 30 minutes | 14 (7%) | 9 (9%) | 5 (5%) | .. |
| 1 hour | 40 (21%) | 20 (20%) | 20 (21%) | .. |
| 2 hours | 39 (20%) | 20 (20%) | 19 (20%) | .. |
| More than 2 hours | 96 (50%) | 46 (47%) | 50 (52%) | .. |
| Previous experience with strength training | | | | .02^c^ |
| Very high | 13 (7%) | 2 (2%) | 11 (11%) | .. |
| High | 38 (20%) | 22 (22%) | 16 (17%) | .. |
| Medium | 92 (47%) | 49 (50%) | 43 (45%) | .. |
| Low | 39 (20%) | 22 (22%) | 17 (18%) | .. |
| Very low | 12 (6%) | 3 (3%) | 9 (9%) | .. |
| Previous experience with exercise therapy (e.g. training with a physiotherapist or sports therapist | | | | .16^c^ |
| Very high | 16 (8%) | 5 (5%) | 11 (12%) | .. |
| High | 46 (24%) | 24 (25%) | 22 (23%) | .. |
| Medium | 85 (44%) | 45 (46%) | 40 (42%) | .. |
| Low | 33 (17%) | 20 (20%) | 13 (14%) | .. |
| Very low | 14 (7%) | 4 (4%) | 10 (10%) | .. |
| Previous experience with hip or knee group training | | | | .40^c^ |
| Yes | 15 (8%) | 6 (6%) | 9 (9%) | .. |
| No | 179 (92%) | 92 (94%) | 87 (91%) | .. |
| **Others** | | | |  |
| Technical affinity [-2; 14]^h^ | 7.5 (2.0) | 7.4 (2.1) | 7.7 (1.9) | .45^b^ |
| Outcome expectation [5; 20]^i^ | 14.4 (3.1) | 14.4 (3.3) | 14.3 (2.9) | .66^b^ |
| Fear of Movement [6; 24]^j^ | 9.6 (3.0) | 9.8 (3.3) | 9.5 (2.6) | .46^b^ |
| ^a^ Data are mean (SD), n (%), or median (IQR). Some percentages might not add to 100% due to rounding.  OA=Osteoarthritis.  ^b^ t-test for independent samples, mean (SD)  ^c^ Chi-Square test for 2*2 Tables  ^d^ Mann-Whitney test, median (IQR)  ^e^ Chi-Square test full degrees of freedom (nominal variables)  ^f^ Knee Osteoarthritis Outcome Score subscale pain scores were categorized into on average no (>87.5 points), mild (>62.5 – 87.5 points), moderate (>37.5 – 62.5 points), severe (>12.5 – 37.5 points) or extreme (0 – 12.5 points) difficulty/pain severity.  ^g^ Chi-Square test for linear trend (ordinal variables)  ^h^ Technical affinity was assessed with the Technical Affinity – Electronic Devices (TA-EG) questionnaire using a 5-point Likert scale with options of “completely applicable”, “rather applicable”, “partly”, “rather not applicable”, and “not applicable at all”. Higher scores reflect a better technical affinity.  ^i^ Outcome expectation was assessed with the Expectation for Treatment Scale (ETS, German version) using a 4-point Likert scale with options of “partially disagree”, “partially agree”, “agree”, and “definitely agree”. Higher scores reflect a higher outcome expectation.  ^j^ Fear of Movement was assessed with the Tampa Scale for Kinesiophobia (TSK-GV, German version) using a 4-point Likert scale with options of “strongly disagree”, “disagree”, “agree”, and “strongly agree”. Higher scores reflect greater fear of movement. | | | | |

# Table A5: Mean (SD) scores on primary and secondary outcome measures for baseline (t0) and after three months (t1), by study group.^a^

|  | **Baseline (t0)** | | **3 months (t1)^b^** | |
| --- | --- | --- | --- | --- |
|  | **re.flex group**  **(n=98)** | **Usual care group**  **(n=96)** | **re.flex group**  **(n=98)** | **Usual care group**  **(n=96)** |
| **Primary outcomes** | | | | |
| Pain (KOOS Pain) | 53.9 (14.4) | 54.9 (12.8) | 63.6 (16.3) | 59.3 (14.6) |
| Physical function (KOOS ADL) | 66.7 (15.1) | 67.7 (15.2) | 75.6 (16.6) | 72.4 (15.2) |
| **Secondary outcomes** | | | | |
| Symptoms (KOOS Symptoms) | 56.0 (17.9) | 57.1 (17.1) | 65.6 (17.5) | 62.1 (17.0) |
| Function in sport and recreation (KOOS Sport/Rec) | 33.6 (17.2) | 33.1 (18.2) | 43.2 (23.0) | 38.3 (20.7) |
| Knee-related quality of life (KOOS QoL) | 32.3 (16.5) | 32.8 (14.2) | 36.5 (17.9) | 34.4 (15.2) |
| Patients Global Assessment | 2.7 (0.8) | 2.8 (0.7) | 2.6 (0.9) | 2.7 (0.7) |
| VR-12 PCS | 37.6 (7.7) | 37.2 (7.9) | 39.4 (8.5) | 36.8 (7.7) |
| VR-12 MCS | 51.5 (10.5) | 50.9 (9.5) | 50.9 (10.7) | 50.6 (10.0) |
| 30s Chair Stand Test | 10.7 (2.4) | 10.6 (2.5) | 12.4 (2.6) | 12.0 (2.6) |
| KOOS=Knee Osteoarthritis Outcome Score (0-100, worse to best); ADL=Activities in Daily Living. Patients Global Assessment is score 1-5 (best to worse). VR-12=Health related quality of life (0-100, worse to best, normalized to a mean value of 50 and SD of 10). PCS=Physical Component Score; MCS=Mental Component Score.  ^b^ Missing values were replaced with multiple imputation method jump to reference | | | | |

# Table A6: Sensitivity analyses for primary and secondary outcomes.^a^

|  | **Analysis** | **n IG (3 months)** | **n CG (3 months)** | **Difference crude**  **(3 months minus baseline)^b^** | **Coefficient b**  **(95% CI)** | **SE** | ***P* value** | **Cohen’s d** |
| --- | --- | --- | --- | --- | --- | --- | --- | --- |
| Pain (KOOS Pain)^c^ | MI (ITT) | 98 | 96 | 10.2  4.5 | 5.3  (1.1 to 9.4) | 2.1 | .01 | 0.38 |
|  | PP | 62 | 91 | 9.5  4.6 | 5.3  (1.2 to 9.5) | 2.1 | .01 | 0.36 |
|  | CC | 88 | 91 | 9.5  4.6 | 5.3  (1.2 to 9.5) | 2.1 | .01 | 0.32 |
|  | LOCF | 98 | 96 | 8.5  4.3 | 3.8  (-0.2 to 7.8) | 2.0 | .06 | 0.28 |
| Physical function  (KOOS ADL)^c^ | MI (ITT) | 98 | 96 | 9.3  4.7 | 4.3  (0.4 to 8.2) | 2.0 | .03 | 0.33 |
|  | PP | 63 | 91 | 10.0  4.7 | 5.8  (2.1 to 9.6) | 1.9 | .003 | 0.42 |
|  | CC | 89 | 91 | 8.9  4.7 | 4.3  (0.3 to 8.2) | 2.0 | .03 | 0.29 |
|  | LOCF | 98 | 96 | 8.1  4.5 | 3.3  (0.5 to 7.1) | 1.9 | .08 | 0.26 |
| Symptoms  (KOOS Symptoms)^c^ | MI (ITT) | 98 | 96 | 10.0  5.0 | 4.5  (0.2 to 8.9) | 2.2 | .04 | 0.31 |
|  | PP | 63 | 91 | 9.2  5.1 | 4.1  (-2.3 to 6.8) | 2.2 | .07 | 0.27 |
|  | CC | 89 | 91 | 10.1  5.1 | 4.5  (0.1 to 8.9) | 2.4 | .04 | 0.29 |
|  | LOCF | 98 | 96 | 9.1  4.9 | 3.8  (-0.3 to 8.0) | 2.1 | .07 | 0.26 |
| Function in sport and recreation  (KOOS Sport/Rec)^c^ | MI (ITT) | 98 | 96 | 10.0  5.2 | 5.0  (-0.3 to 10.2) | 2.7 | .07 | 0.27 |
|  | PP | 63 | 90 | 10.8  5.6 | 5.2  (-0.4 to 10.7) | 2.8 | .07 | 0.29 |
|  | CC | 89 | 90 | 9.9  5.6 | 4.5  (-0.8 to 9.9) | 2.7 | .09 | 0.23 |
|  | LOCF | 98 | 96 | 9.0  5.3 | 3.8  (-1.2 to 8.7) | 2.5 | .13 | 0.20 |
| Knee-related quality of life  (KOOS QoL)^c^ | MI (ITT) | 98 | 96 | 4.4  1.6 | 2.7  (-1.4 to 6.7) | 2.1 | .20 | 0.19 |
|  | PP | 63 | 91 | 4.8  1.9 | 2.9  (-1.2 to 7.1) | 2.1 | .16 | 0.20 |
|  | CC | 89 | 91 | 4.4  1.9 | 2.7  (-1.5 to 6.7) | 2.1 | .21 | 0.17 |
|  | LOCF | 98 | 96 | 4.0  1.8 | 2.1  (-1.7 to 5.9) | 1.9 | .27 | 0.15 |

**Table A6: Sensitivity analyses for primary and secondary outcomes (continued).**

|  | **Analysis** | **n IG (3 months)** | **n CG (3 months)** | **Difference crude**  **(3 months minus baseline)^b^** | **Coefficient b**  **(95% CI)** | **SE** | ***P* value** | **Cohen’s d** |
| --- | --- | --- | --- | --- | --- | --- | --- | --- |
| Patients Global Assessment^d^ | MI (ITT) | 98 | 96 | -0.1  -0.1 | -0.1  (-0.3 to 0.1) | 0.1 | .36 | 0.11 |
|  | PP | 63 | 91 | -0.2  -0.1 | 0.2  (0.0 to 0.5) | 0.1 | .03 | 0.26 |
|  | CC | 89 | 91 | -0.1  -0.1 | 0.1  (-0.1 to 0.3) | 0.1 | .30 | 0.10 |
|  | LOCF | 98 | 96 | -0.1  -0.1 | 0.1  (-0.1 to 0.3) | 0.1 | .37 | 0.09 |
| VR-12 PCS^c^ | MI (ITT) | 98 | 96 | 2.0  -0.4 | 2.6  (0.5 to 4.7) | 1.1 | .01 | 0.31 |
|  | PP | 63 | 91 | 2.6  -0.4 | 3.5  (1.5 to 5.6) | 1.0 | .001 | 0.41 |
|  | CC | 89 | 91 | 1.9  -0.4 | 2.7  (0.6 to 4.7) | 1.1 | .01 | 0.28 |
|  | LOCF | 98 | 96 | 1.7  -0.3 | 2.3  (0.3 to 4.2) | 0.9 | .02 | 0.27 |
| VR-12 MCS^c^ | MI (ITT) | 98 | 96 | -0.6  -0.3 | 0.1  (-2.6 to) 2.4) | 1.3 | .95 | 0.04 |
|  | PP | 63 | 91 | 0.8  0.3 | 1.3  (-3.9 to 1.4) | 1.3 | .35 | 0.12 |
|  | CC | 89 | 91 | -0.5  -0.3 | -0.1  (-2.6 to 2.5) | 1.3 | .95 | 0.03 |
|  | LOCF | 98 | 96 | -0.5  -0.3 | 0.0  (-2.3 to 2.4) | 1.2 | .98 | 0.02 |
| 30s Chair Stand Test^c^ | MI (ITT) | 98 | 96 | 1.7  1.4 | 0.3  (-0.3 to 1.0) | 0.3 | .31 | 0.16 |
|  | PP | 63 | 75 | 1.8  1.5 | 0.4  (-0.3 to 1.1) | 0.4 | .23 | 0.17 |
|  | CC | 89 | 91 | 1.7  1.5 | 0.3  (-0.3 to 0.9) | 0.3 | .34 | 0.12 |
|  | LOCF | 98 | 96 | 1.4  1.1 | 0.3  (-0.2 to 0.8) | 0.3 | .28 | 0.14 |
| ^a^ Differences in change between groups are adjusted for baseline and stratification variables.  IG=intervention group. CG=control group. MI=Multiple imputation. ITT=Intention to treat. PP=per Protocol. CC=Complete Case. LOCF=Least (=baseline) observation carried forward. KOOS=Knee Osteoarthritis Outcome Score (0-100, worse to best). ADL=Activities of Daily Living. Patients Global Assessment is scored 1-5 (best to worse). VR-12=Health related quality of life (0-100, worse to best, normalized to a mean value of 50 and SD of 10). PCS=Physical Component Score. MCS=Mental Component Score.  ^b^ Upper entry intervention group, lower entry control group.  ^c^ For change within groups, positive changes indicate improvement. For difference in change between groups, positive differences favour intervention group (re.flex).  ^d^ For change within groups, negative changes indicate improvement. For difference in change between groups, negative differences favour intervention group (re.flex). | | | | | | | | |

#

# Subgroup analyses

**Table A7: Knee osteoarthritis related concomitant care during the 12-week intervention phase.^a^**

| **Category** | | **CG (usual care)** | **IG (re.flex)** |
| --- | --- | --- | --- |
| Orthopaedic consultation | before and during^b^ | 11 | 9 |
|  | new during^c^ | 6 | 5 |
|  | total number^d^ | 17 | 14 |
| Injection | before and during^b^ | 3 | 1 |
|  | new during^c^ | 3 | 3 |
|  | total number^d^ | 6 | 4 |
| Hard frame knee orthosis | before and during^b^ | 5 | 3 |
|  | new during^c^ | 2 | 0 |
|  | total number^d^ | 7 | 3 |
| **Insoles^e^** | before and during^b^ | 23 | 21 |
|  | new during^c^ | 11 | 5 |
|  | total number^d^ | 34 | 26 |
| Patient information | before and during^b^ | 3 | 1 |
|  | new during^c^ | 7 | 6 |
|  | total number^d^ | 10 | 7 |
| **Physiotherapy^e^** | before and during^b^ | 21 | 14 |
|  | new during^c^ | 6 | 6 |
|  | total number^d^ | 27 | 20 |
| **NSAID (incl. cremes)^e, f^** | before and during^b^ | 11 | 12 |
|  | new during^c^ | 6 | 2 |
|  | total number^d^ | 17 | 14 |
| ^a^ CG=control group. IG=intervention group.  ^b^ Concomitant care was already made use of in the previous 12 months.  ^c^ Concomitant care was initiated during the 12-week intervention phase.  ^d^ Total number of participants who used concomitant care during the intervention period.  ^e^ Those included into the subgroup analysis are highlighted in bold.  ^f^ NSAID= Non-steroidal anti-inflammatory drugs intake on a regular base (weekly or daily). | | | |





**Figure A24: Subgroup analyses for Knee Osteoarthritis Outcome Score (KOOS) Subscale Pain. For difference in change between groups (coefficient b), positive differences favour re.flex group (intervention group).**





**Figure A25: Subgroup analyses for Knee Osteoarthritis Outcome Score (KOOS) subscale Activities in daily living (ADL)*.* For difference in change between groups (coefficient b), positive differences favour re.flex group (intervention group). The only significant interaction was found for physiotherapy (*P*=.03).**

# Adverse Events

Participants of the re.flex group were asked to document any adverse events (AE) occurring during the study period. Participants of the intervention group were further instructed to interrupt the training program in case of any suspicious symptoms, fatigue or excessive pain during the exercise intervention. Minor AEs had to be reported to the responsible study personal within one week (via email, telephone). AEs causing the need for referral to a physician or hospital had to be reported to the responsible study personal immediately. The decision on how to proceed was up to the study physician and the study personal (sports scientist/physiotherapist) and included actions like modifying, pausing, or completely ceasing the training program. In case of persistent complaints, patients were presented again to the physician to initiate further medical interventions, if necessary.

**Table A8: Adverse Event Reporting*.*^a^**

| **ID^b^** | **Harm** | **Type^c^** | **Exp^d^** | **Link^e^** | **MC^f^** | **CoI^g^** |
| --- | --- | --- | --- | --- | --- | --- |
| 006 | Back and groin pain | AE | UE | S | no | stop |
| 087 (DO) | Increased pain due to device-initiated overload (repetition count failure due to movement restrictions of OA knee) | AE | UE | S | no | stop |
| 140 | Increasing knee pain associated with specific exercise | AE | UE | S | yes | stop |
| 063 | Severe pain in the knee joint during rest. Irritated knee. | AE | EE | S | no | pause |
| 129 | Pain during specific exercises | AE | EE | S | no | mod |
| 025 | Increasing buttock/sciatica pain during specific exercises, sometimes also at night | AE | UE | L | yes | stop |
| 051 (DO) | Activated knee OA with effusion | AE | UE | L | yes | stop |
| 107 (DO) | Increasing pain at the sacroiliac joint due to device-initiated overload (repetition count failure due to movement restrictions of OA knee) | AE | UE | L | no | stop |
| 035 | Pain like muscle strain after the first exercise session (short-term) | AE | EE | L | no | none |
| 152 | Knee inflammation | AE | UE | L | no | pause |
| 123 | Increasing knee joint pain | AE | EE | P | yes | stop |
| 010 | Increased knee pain | AE | EE | P | no | pause |
| 003 | Covid-19 and Bursitis at the hip | AE | UE | U | yes | stop |
| 020 | Hayfever, associated with rheumatic flare | AE | UE | U | yes | stop |
| 130 (DO) | Pain in back and knee. According to report of patient, no link to intervention. Wants to cease study. | AE | NA | U | no | stop |
| 019 | Meniscal surgery due to a prior injury before the start of the study | SAE | UE | U | yes | pause |
| 038 | Hospital stay because of gallstones | SAE | NA | N | yes | pause |
| 100 (DO) | Stroke (Trans Ischemic attack) | SAE | NA | N | yes | stop |
| 110 | Knee arthroscopy after advice from external physician in the context of usual care | SAE | NA | N | yes | pause |
| 119 | Hospital stay because of heart catheter | SAE | NA | N | yes | pause |
| ^a^ Reports were classified into AE=adverse event, and SAE=serious adverse events (events related to death, life-threatening illness or injury, in-patient hospitalization). They were further classified into EE=expected, and UU=unexpected events, and the link to intervention was differentiated into S=sure, L=likely, P=possible, U=unlikely, N=no. Actions taken were classified into need (=yes)/no need (=no) for immediate medical care (e.g. referral to orthopaedist, physiotherapy, medication) and change of intervention modalities (e.g. mod=modification, pause=pausing, stop=stopping, none=no change).  ^b^ DO=Drop-out; ^c^ Type of event; ^d^ Expectation: NA=not applicable/no link; ^e^ Link to intervention; ^f^ MC=Medical care; ^g^ Change of intervention | | | | | | |

# Exercise-related pain

Exercise pose, duration, load, wrong movement execution, or exercise-related muscle soreness were mentioned by 24 (27%) participants, 10 (11%) participants mentioned symptoms other than osteoarthritis causing pain, 7 (8%) participants related pain to prevalent knee osteoarthritis, and 12 (14%) participants named other or unknown reasons.

Related adaptations of the training behaviour were reported by 22 participants of which 12 (13.5%) modified exercises, 7 (8%) stopped training, 3 (3%) focused on better exercise execution and 1 (1%) took medication to manage exercises. Table 9 gives an overview on pain incidence, frequency, duration, and intensity.

**Table A9: Frequency of self-reported exercise-related pain during exercise (at three months, n [% intervention group]).**

|  | |  | |  | | |  |  | |  | |
| --- | --- | --- | --- | --- | --- | --- | --- | --- | --- | --- | --- |
| **Incidence** | | | **Frequency** | | | **Duration** | | | **Intensity^a^** | | |
| Yes | 53 (52%) | | Always | | 4 (4%) | Min to few h | | 29 (30%) | 2 | | 16 (16%) |
|  |  | | Frequently | | 7 (7%) | 1 day | | 13 (13%) | 4 | | 11 (11%) |
|  |  | | Sometimes | | 39 (40%) | ≤ 1 week | | 5 (5%) | 6 | | 16 (16%) |
|  |  | | Once | | 3 (3%) | > 1 week | | 6 (6%) | 8 | | 9 (9%) |
|  |  | |  | |  |  | |  | 10 | | 1 (1%) |
| No pain | 36 (37%) | | No pain | | 36 (37%) | No pain | | 36 (37%) | No pain | | 36 (37%) |
| Missing^b^ | 0 (0) | | Missing | | 0 (0) | Missing | | 0 (0) | Missing | | 0 (0) |
| DO t1 | 9 (9%) | | DO t1 | | 9 (9%) | DO t1 | | 9 (9%) | DO t1 | | 9 (9%) |
| ^a^ Likert scale scored 0 (no pain), 2 (little pain), 4 (moderate pain), 6 (much pain), 8 (very much pain) or 10 (highest imaginable pain).  ^b^ Missing data do not include drop-outs (DO) at three months (DO t1) of the intervention group (n = 9). | | | | | | | | | | | |

# Table A10: Logfile data for week 1, week 12, and overall.

|  | **Patients of the re.flex group** | | |
| --- | --- | --- | --- |
|  | **Week 1** | **Week 12** | **Overall** |
| Attrition rate (%)^b, c^ | 2 | 28 | .. |
| Exercise session adherence (%)^c, d^ | 94 | 65 | 77 |
| Exercise repetition adherence (%), mean (SD)^c, e^ | 91 (27) | 63 (47) | 74 (43) |
| Active time (min), mean (SD)^f^ | 21 (5)^g^ | 11 (4)^h^ | 18 (6)^i^ |
| ^a^ Missing date were not replaced with multiple imputation.  ^b^ Attrition rate is the percentage of participants who did not log into the re.flex app at least once a week.  ^c^Data refer to all patients of the re.flex group (n=98); 12 weeks with 3 exercise sessions per week.  ^d^ Exercise session adherence is the percentage of conducted exercise sessions relative to the overall number of prescribed exercise sessions during the study period.  ^e^ Exercise repetition adherence is the percentage of all valid repetitions in percent with a maximum value of 100%.  ^f^ Active time is the crude time for exercising with the sensor-equipped leg without login, calibration, video watching, and feedback on pain and exertion.  ^g^ Data refer to 276 exercise sessions.  ^h^ Data refer to 192 exercise sessions.  ^i^ Data refer to 2706 exercise sessions. | | | |

# Table A11: Perceived exertion and pain outcomes before, during, and after exercising.

|  | **Values, mean (SD)** | **Values, median (IQR)** | **Range** |
| --- | --- | --- | --- |
| Perceived exertion during exercise^a, b^ | 3.5 (1.1) | 3.0 (1.0) | 0.0-10.0 |
| Perceived exertion after exercise^a, b^ | 2.8 (1.7) | 3.0 (0.0) | 0.0-10.0 |
| Delta pain^a^ | 0.3 (1.2) | 0.0 (0.0) | -8.0-8.0 |
| Perceived pain during exercise^a, c^ | 0.9 (1.5) | 0.0 (2.0) | 0.0-10.0 |
| ^a^ Data out of 2705 exercise sessions.  ^b^ 10-point scale from 0 (no exertion at all) to 10 (maximum conceivable exertion).  ^c^ 10-point scale from 0 (no pain) to 10 (highest imaginable pain). | | | |

# Table A12: Patient satisfaction of the re.flex app and treatment satisfaction.

|  | **re.flex group (n=89)** | **Usual care group (n=91)** |
| --- | --- | --- |
| Patient satisfaction (ZUF-8)^a^, mean (SD) | 24.9 (5.0) | .. |
| Treatment satisfaction, n (%) |  |  |
| Very satisfied | 23 (25.8) | 6 (6.6) |
| Satisfied | 33 (37.1) | 14 (15.4) |
| Neither/nor satisfied | 24 (27.0) | 59 (64.8) |
| Unsatisfied | 7 (7.9) | 11 (12.1) |
| Very unsatisfied | 2 (2.2) | 1 (1.1) |
| ^a^ ZUF-8=Patient satisfaction questionnaire (8-32, higher values indicating higher patient satisfaction with the app). | | |

# References

1. Fernandes L, Hagen KB, Bijlsma JW, Andreassen O, Christensen P, Conaghan PG, et al. EULAR recommendations for the non-pharmacological core management of hip and knee osteoarthritis. Ann Rheum Dis. 2013;72(7):1125-35.

2. McAlindon TE, Bannuru RR, Sullivan MC, Arden NK, Berenbaum F, Bierma-Zeinstra SM, et al. OARSI guidelines for the non-surgical management of knee osteoarthritis. Osteoarthritis Cartilage. 2014;22(3):363-88.

3. Garber CE, Blissmer B, Deschenes MR, Franklin BA, Lamonte MJ, Lee IM, et al. American College of Sports Medicine position stand. Quantity and quality of exercise for developing and maintaining cardiorespiratory, musculoskeletal, and neuromotor fitness in apparently healthy adults: guidance for prescribing exercise. Med Sci Sports Exerc. 2011;43(7):1334-59.

4. Haupt G, Janßen P, Krauß I, Steinhilber B. Das Tübinger Hüftkonzept. Essen: Verlag hellblau; 2014. p. 1-176.

5. Krauss I, Steinhilber B, Haupt G, Miller R, Martus P, Janssen P. Exercise therapy in hip osteoarthritis - a randomized controlled trial. Dtsch Arztebl Int. 2014;111(35/36):592-9.

6. Steinhilber B, Haupt G, Miller R, Boeer J, Grau S, Janssen P, et al. Feasibility and efficacy of an 8-week progressive home-based strengthening exercise program in patients with osteoarthritis of the hip and/or total hip joint replacement: a preliminary trial. Clinical rheumatology. 2012;31(3):511-9.

7. Merk J, Horstmann T, Krauss I, Danzinger B, Ziegler C, Belzl H. Sporttherapie für Patienten mit Kniearthrose oder Knieendoprothese-Konzept, Inhalte und Evaluation der Tübinger Kniesportgruppe. Zeitschrift für Physiotherapeuten. 2005(57):262--73.

8. Dieter V, Janssen P, Krauss I. Efficacy of the mHealth-Based Exercise Intervention re. flex for Patients With Knee Osteoarthritis: Pilot Randomized Controlled Trial. JMIR mHealth and uHealth. 2024;12(1):e54356.

9. Rolfson O, Wissig S, van Maasakkers L, Stowell C, Ackerman I, Ayers D, et al. Defining an international standard set of outcome measures for patients with hip or knee osteoarthritis: consensus of the international consortium for health outcomes measurement hip and knee osteoarthritis working group. Arthritis care & research. 2016;68(11):1631-9.

10. Karrer K, Glaser C, Clemens C, Bruder C. Technikaffinität erfassen–der Fragebogen TA-EG. Der Mensch im Mittelpunkt technischer Systeme. 2009;8:196-201.

11. Barth J, Kern A, Lüthi S, Witt CM. Assessment of patients’ expectations: development and validation of the Expectation for Treatment Scale (ETS). BMJ open. 2019;9(6):e026712.

12. Rusu AC, Kreddig N, Hallner D, Hülsebusch J, Hasenbring MI. Fear of movement/(Re) injury in low back pain: confirmatory validation of a German version of the Tampa Scale for Kinesiophobia. BMC musculoskeletal disorders. 2014;15(1):1-9.

13. Kessler S, Lang S, Puhl W, Stöve J. Der Knee Injury and Osteoarthritis Outcome Score: Ein Funktionsfragebogen zur Outcome-Messung in der Knieendoprothetik. Z Orthop Grenzgeb. 2003;141(3):277-82.

14. Roos EM, Lohmander LS. The Knee injury and Osteoarthritis Outcome Score (KOOS): from joint injury to osteoarthritis. Health and quality of life outcomes. 2003;1(1):1-8.

15. Schnitzer TJ, Easton R, Pang S, Levinson DJ, Pixton G, Viktrup L, et al. Effect of tanezumab on joint pain, physical function, and patient global assessment of osteoarthritis among patients with osteoarthritis of the hip or knee: a randomized clinical trial. Jama. 2019;322(1):37-48.

16. Iqbal S, Rogers W, Selim A. The Veterans RAND 12 Item Health Survey (VR-12): What It Is and How It Is Used. Bedford, Mass, Veterans Administration Medical Center. Center for Health Quality, Outcomes and Economic Research, and Boston University School of Public Health, Center for the Assessment of Pharmaceutical Practices. 2007.

17. Buchholz I, Feng Y-S, Buchholz M, Kazis LE, Kohlmann T. Translation and adaptation of the German version of the Veterans Rand—36/12 Item Health Survey. Health and quality of life outcomes. 2021;19:1-16.

18. Angst F, Benz T, Lehmann S, Aeschlimann A, Angst J. Multidimensional minimal clinically important differences in knee osteoarthritis after comprehensive rehabilitation: a prospective evaluation from the Bad Zurzach Osteoarthritis Study. RMD Open. 2018;4(2):e000685-e.

19. Centers for Disease Control and Prevention. Assessment 30-Second Chair Stand. 2017 [accessed 03.05.2024]. Available from: https://www.cdc.gov/steadi/pdf/STEADI-Assessment-30Sec-508.pdf.

20. Zhou L, Bao J, Setiawan IMA, Saptono A, Parmanto B. The mHealth APP usability questionnaire (MAUQ): development and validation study. JMIR mHealth and uHealth. 2019;7(4):e11500.

21. Schmidt J, Wittmann WW, editors. Fragebogen zur messung der patientenzufriedenheit. Diagnostische verfahren in der psychotherapie Göttingen: Hogrefe; 2002.

22. Dieter V, Martus P, Janssen P, Krauss I. Evaluation of a 12-week app-guided exercise intervention in patients with knee osteoarthritis (re.flex): a study protocol for a randomized controlled trial. BMC Digital Health. 2023;1(1):43.

23. Verhagen AP, Ferreira M, Reijneveld-van de Vendel EAE, Teirlinck CH, Runhaar J, van Middelkoop M, et al. Do we need another trial on exercise in patients with knee osteoarthritis: No new trials on exercise in knee OA. Osteoarthritis Cartilage. 2019;27(9):1266–9

24. Angst F, Benz T, Lehmann S, Aeschlimann A, Angst J. Multidimensional minimal clinically important differences in knee osteoarthritis after comprehensive rehabilitation: a prospective evaluation from the Bad Zurzach Osteoarthritis Study. RMD Open. 2018;4(2):e000685-e.

25. Mills KAG, Naylor JM, Eyles JP, Roos EM, Hunter DJ. Examining the Minimal Important Difference of Patient-reported Outcome Measures for Individuals with Knee Osteoarthritis: A Model Using the Knee Injury and Osteoarthritis Outcome Score. The Journal of Rheumatology. 2016;43(2):395-404.
